# From Hub Proteins to Hub Modules: The Relationship Between Essentiality and Centrality in the Yeast Interactome at Different Scales of Organization

**DOI:** 10.1371/journal.pcbi.1002910

**Published:** 2013-02-21

**Authors:** Jimin Song, Mona Singh

**Affiliations:** Department of Computer Science and Lewis-Sigler Institute for Integrative Genomics, Princeton University, Princeton, New Jersey, United States of America; Harvard Medical School, United States of America

## Abstract

Numerous studies have suggested that hub proteins in the *S. cerevisiae* physical interaction network are more likely to be essential than other proteins. The proposed reasons underlying this observed relationship between topology and functioning have been subject to some controversy, with recent work suggesting that it arises due to the participation of hub proteins in essential complexes and processes. However, do these essential modules themselves have distinct network characteristics, and how do their essential proteins differ in their topological properties from their non-essential proteins? We aimed to advance our understanding of protein essentiality by analyzing proteins, complexes and processes within their broader functional context and by considering physical interactions both within and across complexes and biological processes. In agreement with the view that essentiality is a modular property, we found that the number of intracomplex or intraprocess interactions that a protein has is a better indicator of its essentiality than its overall number of interactions. Moreover, we found that within an essential complex, its essential proteins have on average more interactions, especially intracomplex interactions, than its non-essential proteins. Finally, we built a module-level interaction network and found that essential complexes and processes tend to have higher interaction degrees in this network than non-essential complexes and processes; that is, they exhibit a larger amount of functional cross-talk than their non-essential counterparts.

## Author Summary

Network analyses of large-scale interactomes have been a great aid in advancing our understanding of cellular functioning and organization. Here, we examine one of the most basic and intensely-studied structure-to-function relationships observed in cellular networks: that between the number of interactions a protein has and its tendency to be essential. We develop a new computational framework to systematically analyze essential proteins within their cellular context by explicitly incorporating functional information. We apply this framework to the yeast interactome and demonstrate that the previously observed positive relationship between interaction degree and essentiality is largely due to intramodular interactions. Further, essentiality appears to be a modular property of protein complexes and not more broadly of biological processes. Within an essential complex, its essential proteins tend to have more interactions, especially intra-complex interactions, than its non-essential proteins. Finally, in a computationally inferred module-level interaction network, essential complexes and processes tend to have higher interaction degrees than their non-essential counterparts. In summary, we show a relationship between connectivity and essentiality not only at the protein level, but also within modules and at the module level, with complexes and processes that are essential tending to interact with many functional groups.

## Introduction

High-throughput experimental approaches for determining protein interactions have resulted in large-scale cellular networks for numerous organisms. Graph-theoretic analyses of these networks have been a great aid in advancing our understanding of cellular functioning and organization (review, [1]). One of the most fundamental discoveries is that there is a strong relationship between the topological characteristics of cellular networks and their underlying functioning. For example, cellular networks consist of tightly clustered groups of interacting proteins, and these proteins work together as protein complexes or biological processes to achieve specific biological functions [2]–[7]. An orthogonal decomposition reveals that there are recurring and over-represented topological and functional patterns within larger cellular networks, and these network motifs [8], [9] and network schemas [10] can be associated with dynamic regulatory properties and shared mechanisms of functioning. Here, we revisit perhaps the most basic structure-to-function relationship that has been proposed for cellular networks—that between the number of interactions a protein has and its overall functional importance.

The importance of a gene to a cell or an organism can be quantitatively measured by considering the phenotypic effects of gene deletion or disruption. Experimental studies in the baker's yeast *S. cerevisiae* have demonstrated that approximately 19% of its proteins are essential; that is, the deletion of these proteins results in cell death, even in optimal growth conditions [11], [12]. Early computational analysis of the yeast *S. cerevisiae* protein-protein physical interaction network revealed a scale-free topology, where a few “hub” proteins have many interactions, and also showed that hub proteins are more likely to be essential than other proteins [13]. Numerous subsequent studies have confirmed this centrality-lethality relationship, not only in yeast [14]–[19] but also in other organisms [20]. On the other hand, the relationship has been observed to be weak in networks consisting of interactions determined via high-throughput yeast two-hybrid experiments while stronger in other types of networks [16], [18], [19], and it has been proposed that, in yeast two-hybrid networks, the observed relationship is due to study bias favoring the determination of interactions of essential proteins [21]. Nevertheless, the positive correlation between protein interaction degree and essentiality is generally accepted, with numerous reasons proposed in the literature to explain this relationship.

Initial work suggested that high-degree proteins may be essential due to their role in interaction network connectivity [13]; however, this is unlikely to be the case as it was subsequently shown that non-essential hubs are just as important as essential hubs for maintaining connectivity, and that essentiality is better correlated with local, rather than global, measures of connectivity in protein-protein interaction networks [17], [18]. It was alternatively proposed that essentiality is a property of interactions [22]. That is, there are essential protein interactions, without which an organism cannot survive, and these are randomly distributed across the network; hubs then tend to be essential as they are more likely to participate in essential interactions. However, this model implies that the probabilities that two non-interacting proteins are essential are independent of each other, and this is not the case [18]. Instead, Zotenko et al. [18] argued that the correlation between degree and essentiality is due to the participation of essential proteins in essential functional modules consisting of groups of densely clustered and functionally related proteins. They further showed that the essentiality of hubs that are not in these computationally extracted modules are only weakly correlated with degree [18]. Indeed, it had previously been found that essential proteins tended to be densely connected to each other [15] and concentrated in complexes 23,24, suggesting that essentiality is a modular property rather than a property of individual proteins. Building upon this, it has been argued that essential complexes tend to be large, and thus proteins within them have a larger number of interactions, and that this explains why hubs tend to be essential [19].

While there is substantial evidence that essentiality is a modular property in protein-protein interaction networks, it is also clear that complexes and processes do not consist entirely of essential or non-essential proteins. Do essential proteins within an essential complex or process differ from the non-essential ones? Further, not all complexes and processes contain essential proteins. Do such essential modules have distinctive roles in cellular networks? In this paper, we aimed to discover whether, within modules, their essential and non-essential proteins differ in their interaction properties, and at a more global scale, whether essential and non-essential modules differ in their network-level properties. To accomplish this, we developed a computational framework that incorporates information about functional modules within the context of network analysis techniques. To uncover general and robust principles, we performed our analysis on three types of *S. cerevisiae* protein-protein interaction networks and considered functional modules derived from protein complexes as well as Gene Ontology (GO) biological process annotations [25] at different levels of resolution. Further, to address the issue of study bias, we performed our analysis on additional networks which removed interactions determined in small-scale experiments.

We began by re-examining the relationship between protein essentiality and network modularity. We hypothesized that if essentiality is a modular property, as has been proposed previously [18], then a protein's intramodular physical interaction degree should be a better predictor of a protein's essentiality than its intermodular physical interaction degree. To test this, we utilized biological process functional annotations of proteins and classified physical interactions into intraprocess interactions within processes and interprocess interactions between processes. We found that essential proteins tend to have many interactions with proteins within the same functional modules and that the intraprocess interaction degree is more correlated with essentiality than overall degree. Further, we found that the relationship between overall degree and essentiality is significantly weakened when controlling for intramodular degree, but is not as affected when controlling for intermodular degree. Thus, we show in a direct and simple manner that, for many essential proteins, their essentiality is likely to be a consequence of their participation within essential modules consisting of functionally similar proteins.

To further ascertain whether the modularity of essential proteins is due to their roles within essential protein complexes or more generally within essential biological processes, we repeated this analysis while first exclusively focusing on proteins within protein complexes and next focusing only on proteins that are not within known protein complexes. We found that most essential proteins with many intraprocess interactions in fact participate in essential protein complexes or in essential biological processes that include one or more protein complexes; that is, the modularity of protein essentiality appears to be a consequence of protein complexes, not more broadly of biological processes.

Next, we examined complexes that contain essential proteins, and compared their essential and non-essential proteins. We reasoned that if the relationship between essentiality and interaction degree for proteins within these complexes is entirely a consequence of the complexes themselves being essential, then essential and non-essential proteins within the same complex should not differ with respect to degree. On the contrary, we found that essential proteins tend to have more interactions, particularly intracomplex interactions, than their non-essential counterparts within protein complexes. That is, while essentiality appears to be a modular property, the degree of a protein is associated with essentiality within essential complexes; we hypothesize that these essential proteins may play a more important role in maintaining the structure and/or function of complexes.

Finally, we analyzed modules containing essential proteins within the context of other functional modules. We inferred significant “cross-talks” between protein complexes and biological processes and used them to build module-level networks, in which two complexes or processes are linked if they have an enriched number of physical interactions between them. Using these module-level networks, we uncovered that functional modules with essential proteins tend on average to have higher degree; that is, degree in the module-level network is positively correlated with module essentiality.

Overall, by considering proteins within the functional context of the yeast interactome, we give evidence that there is a relationship between essentiality and network topology at different levels of cellular organization: at the protein level, within protein complexes, and also more globally at the module level, with complexes and processes that are essential tending to interact with more functional groups.

## Results

We analyzed 5640 proteins that were tested for essentiality [12] in the context of several large-scale *S. cerevisiae* protein physical interaction datasets; each of these networks captures different features of biological interactions. The first network is a *Direct* interaction network, where an interaction between two proteins corresponds to a direct physical contact; this network includes interactions determined by the yeast two-hybrid method among other types of approaches (see Materials and Methods). Next, we considered a *Pull-down* network, where an interaction between two proteins corresponds to their being members in the same multiprotein complex. Third, we considered the *Full* network consisting of all physical interactions in BioGRID [26]; in this case, the interactions can represent either direct or indirect interactions. In the main body of the paper, we primarily report our results on the *Direct* interaction network, which contains 4031 proteins (898 of which are essential) and 15,073 interactions. All the analysis described below is also performed on the *Pull-down* and *Full* networks (see Table S1) and reported in full in the Supplementary Material. We also considered additional networks where interactions determined in small-scale experiments were removed; this analysis is outlined in the section on high-throughput networks below, with detailed figures given in the Supplementary Material.

### Categorizing interactions as intramodular or intermodular

For a given interaction network, we labeled protein interactions as either “intramodular,” “intermodular” or neither using two sources of functional data. In particular, we utilized yeast protein complex data compiled in [27] and Gene Ontology (GO) Biological Process (BP) annotations [25]. Thus, intramodular interactions can arise from either intracomplex or intraprocess interactions, and intermodular interactions arise as either intercomplex or interprocess interactions; we will separately consider both types of intramodular and intermodular interactions. For protein complex data, “intracomplex” interactions are between all pairs of proteins that participate in a shared complex and “intercomplex” interactions are between pairs of proteins that are each found in at least one complex but are never found in the same complex.

It is more complicated to characterize interactions as intramodular or intermodular using GO BP terms, as the terms are hierarchically related and annotate different numbers of proteins, with some very general terms. To get only informative and specific terms, we considered GO BP terms that annotate at most 50 proteins in the yeast proteome. An interaction is unannotated unless both proteins are annotated with any one of these specific GO BP terms. An interaction is “intraprocess” if it is between two proteins sharing one of these specific BP terms. If two proteins with an interaction are annotated with specific GO BP terms but do not share any of them, the interaction is “interprocess.” We note that while physical interactions are largely thought of as “within process,” especially as compared to other types of interactions [28], a significant fraction of physical interactions are interprocess (Table S2); this is true even as the threshold for choosing specific terms is increased.

### Intraprocess interactions are a main factor in the relationship between protein essentiality and interaction degree

**Figure 1:**
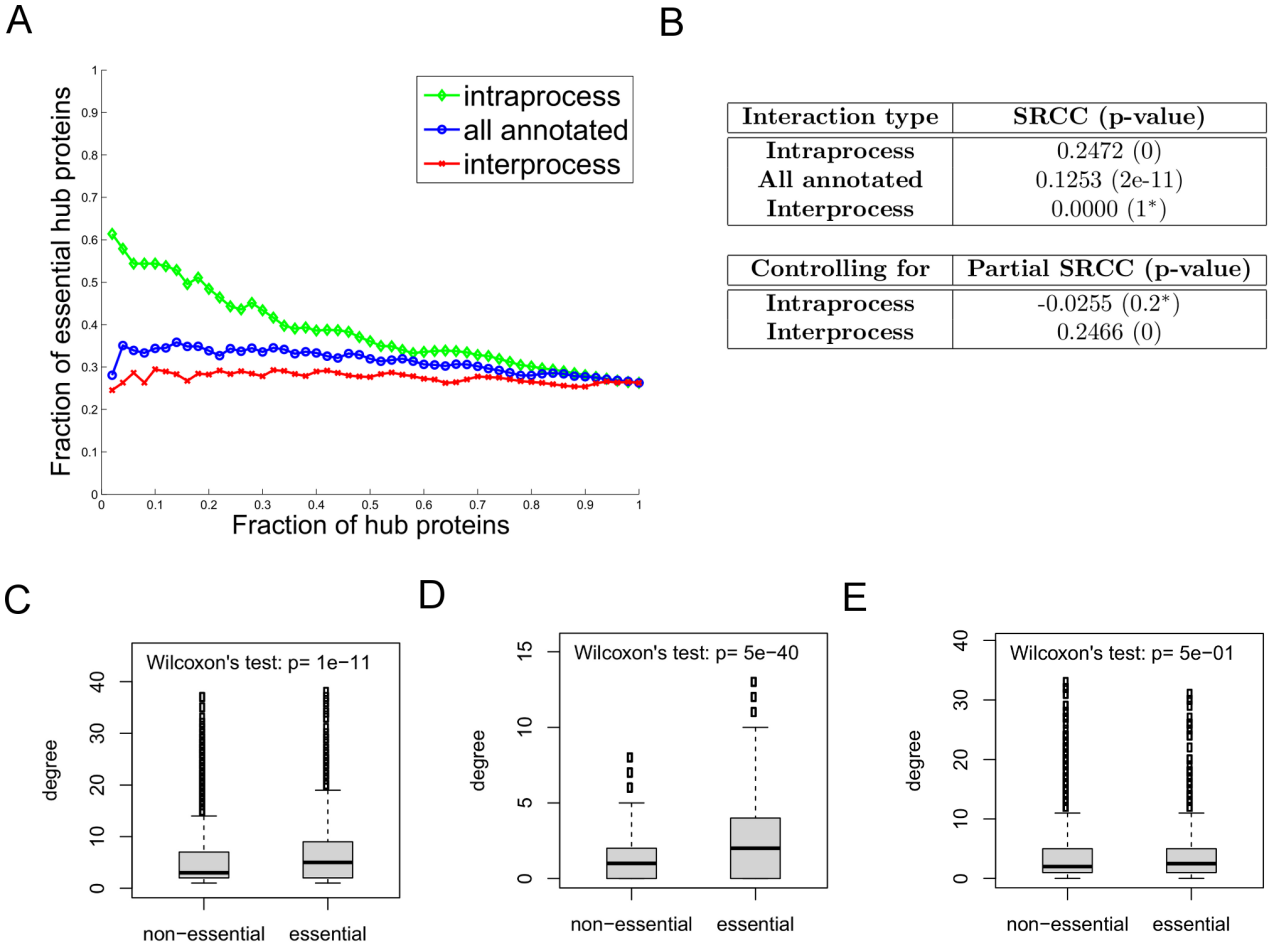
The intraprocess interaction degree is more correlated with protein essentiality than the overall interaction degree for proteins in the *Direct* network, when interactions are categorized with specific BP terms, each of which annotates at most 50 proteins. (**a**) The fraction of essential proteins among hub proteins as more proteins are considered hub proteins; proteins are added in groups of 50 in a non-increasing order of the interaction degree. This fraction is highest for intraprocess degree (green), followed by all annotated degree (blue) and then by interprocess degree (red). (**b**) The correlations measured by SRCCs between essentiality and either intraprocess, all annotated or interprocess degree. The SRCC is highest between essentiality and intraprocess degree. The partial correlation is also computed between all annotated degree and essentiality when controlling for either intraprocess or interprocess degree. Starred 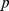-values indicate those with values 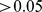. (**c**)**–**(**e**) The degree distribution of non-essential proteins is compared to that of essential proteins for (c) all annotated, (d) intraprocess, and (e) interprocess degree, respectively. In each box plot, the horizontal bar within a box corresponds to the median of the distribution; the two ends of the box indicate the first and third quartiles; and the small circles show outliers within the 2–98th percentile range. The significance of the difference between the two degree distributions is measured by the Wilcoxon rank sum test.

As a first step towards relating protein essentiality to network modularity, for each protein, we computed its number of intraprocess interactions, interprocess interactions, and total annotated interactions. We then considered each of the intraprocess, interprocess and total annotated interaction degrees in turn, and ordered all proteins from high to low degrees with respect to it. As we varied a threshold for the number of proteins considered, we computed the fraction of essential proteins in the “high degree” or “hub” set. Over a large range of thresholds, the high degree proteins, as ranked by intraprocess degree, have a higher fraction of essential proteins than the high degree proteins as ranked by either total annotated degree or interprocess degree (Figures 1 (a), S1 (a) and S2 (a)). For the *Pull-down* and *Full* networks, the fraction of essential proteins tends to decrease as the threshold for intraprocess, interprocess, or total degree is lowered. In the *Direct* network (Figure 1 (a)), this trend is only true for intraprocess interaction degree and is notably not true for total degree; this is consistent with previous work showing that the relationship between essentiality and overall interaction degree is weak in networks consisting of interactions determined by yeast two-hybrid [16], [18], [21].

To further quantify the correlation between essentiality and degree, we used the Spearman's rho rank correlation coefficient (SRCC) [29] (Figures 1 (b), S1 (b) and S2 (b)), and found that the SRCC is highest for intraprocess degree (0.25 for *Direct*, and 0.35 for the other two networks), and much lower for interprocess degree (0 for *Direct*, 0.22 for *Pull-down* and 0.21 for *Full*). We note that since protein essentiality is a binary value and thus there are many tied values, it is not possible for the SRCC to achieve a value of 1. For example, the SRCC between essentiality and all annotated degree in the *Direct* network could at most reach a maximum value of 0.7680 (i.e., the case where the essentiality values for the proteins are swapped so that all essential proteins have higher degrees than all non-essential proteins). Next, to disentangle the contributions of intraprocess and interprocess degree to the observed correlations, we computed partial correlations between essentiality and all annotated interactions, when controlling for intraprocess and interprocess degree. For the three networks we found that when controlling for intraprocess degree, the SRCC between total degree and essentiality notably diminished, whereas when controlling for interprocess degree, the SRCC remained high (Figures 1 (b), S1 (b) and S2 (b)), and even increased for the *Direct* network.

As another way of looking at the difference between intraprocess and interprocess interaction degree, we compared the degree distributions of essential proteins and non-essential proteins (Figures 1, S1 and S2 (c)–(e)) using the Wilcoxon rank sum test. For comparing degree distributions, we included all proteins with at least one annotated interaction; these proteins may have zero intraprocess or interprocess interactions. Since the same number of proteins are considered when comparing total, intraprocess, or interprocess degree (Figures 1, S1 and S2 (c)–(e)), the 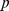-values given are comparable. The difference in the number of interactions between essential and non-essential proteins is much more significant when only intraprocess interactions are considered (Figures 1 (d), S1 (d) and S2 (d)), as compared with the case when all annotated interactions are considered (Figures 1 (c), S1 (c) and S2 (c)) or when only interprocess interactions are considered (Figures 1 (e), S1 (e) and S2 (e)).

As an alternative to categorizing all annotated interactions as either interprocess or intraprocess, we also considered the case where interactions are weighted according to the semantic similarity [30] between the functional terms annotating the two proteins. This weight is in the range of 0 and 1 with proteins sharing highly specific functional terms getting higher scores (see Materials and Methods for more details). Thus, the semantic similarity between two interacting proteins is a continuous measure of the “intramodularity” of the interaction. Then, the semantic similarity degree of a protein is defined as the sum of semantic similarity of interactions. Across the *Direct*, *Pull-down* and *Full* networks, we find that there is a stronger correlation between essentiality and degree when all interactions are weighted with semantic similarity than when they are just counted (Figure S3). In other words, proteins having many interactions within a similar functional context are more likely to be essential than proteins having many interactions. Altogether, a range of computational analyses shows that a large portion of the observed relationship between essentiality and interaction degree can be explained when considering just intraprocess interactions.

### The correlation between intramodular degree and protein essentiality is largely due to complexes, not processes

Having shown the strong correlation between intraprocess interaction degree and essentiality, we sought to characterize the contribution of intracomplex interactions. In particular, previously it had been observed that essential proteins tend to be clustered together within essential protein complexes [18], [24]. Thus, we hypothesized that having intracomplex physical interactions for a protein is more important for predicting its essentiality than having other types of physical interactions. That is, as we have defined them, functional modules can be comprised either of protein complexes or biological processes corresponding to GO BP terms. In the previous section, we utilized modules derived from BP terms. We next focus on modules derived from protein complexes, as compiled in [27]. We begin by observing that complexes as a whole are enriched in essential proteins. In particular, whereas 18.60% (or 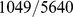) of proteins are essential in the yeast genome, 37.54% (or 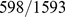) are essential when considering proteins involved in the set of complexes we are considering. In fact, 57.01% (or 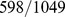) of all essential proteins are involved in protein complexes, even though only 28.24% (or 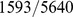) of proteins take part in our set of complexes. Thus, any conclusions arising from the analysis of protein complexes is based on the interaction properties of a significant fraction of essential proteins.

**Figure 2:**
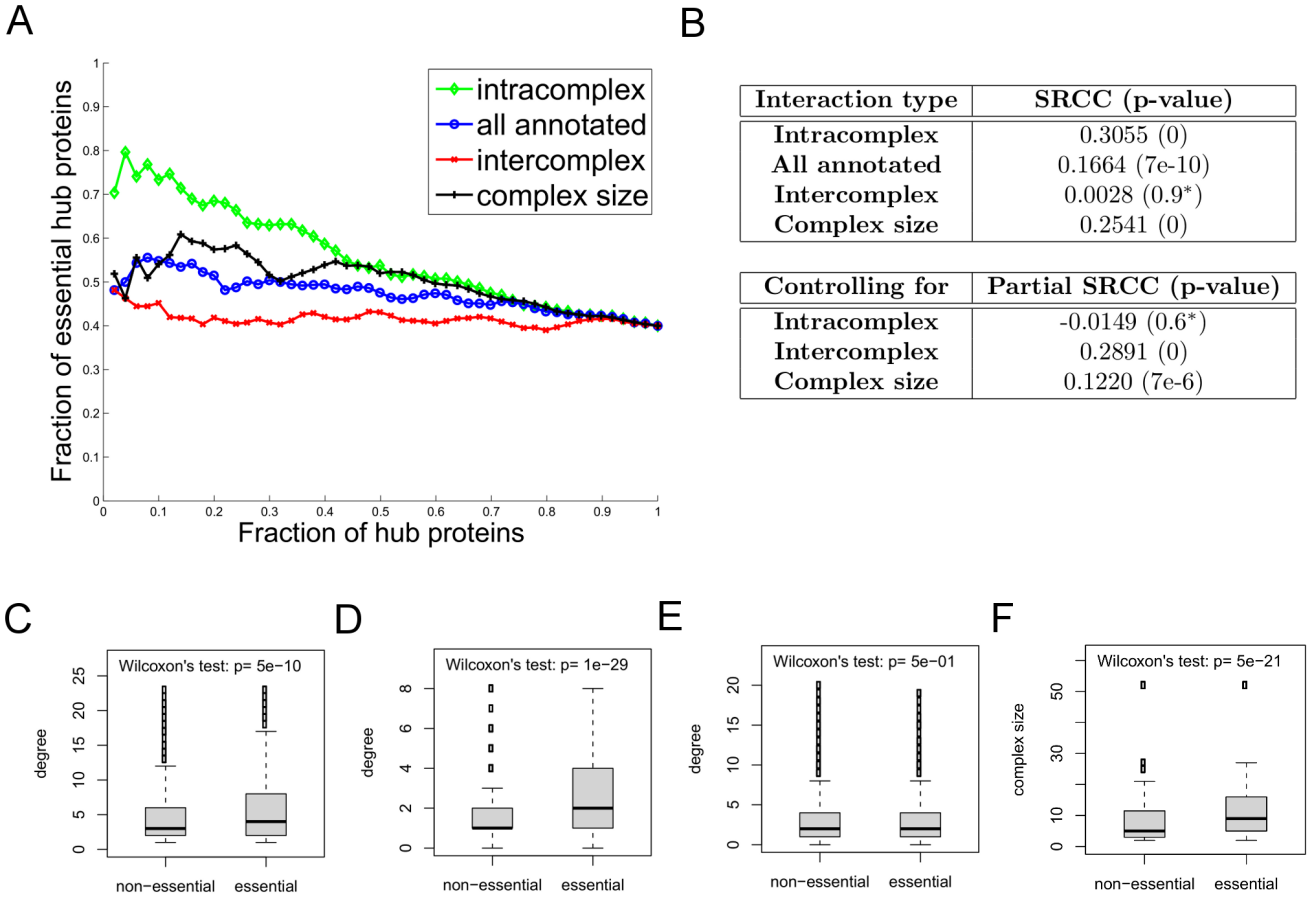
The intracomplex interaction degree is more correlated with protein essentiality than the overall interaction degree for proteins in the *Direct* network, when interactions are categorized using protein complexes. (**a**) The fraction of essential proteins among hub proteins as more proteins are considered hub proteins; proteins are added in groups of 50 in a non-increasing order of the interaction degree or the size of the largest complex to which the protein belongs. The relationship between protein essentiality and interaction degree is shown for intracomplex interactions (green), all annotated interactions (blue) and intercomplex interactions (red). The relationship between protein essentiality and complex size is also shown (black). (**b**) The correlations measured by SRCCs between essentiality and either intracomplex degree, all annotated degree, intercomplex degree, or complex size. The SRCC is highest between essentiality and intracomplex degree. The partial correlation is also computed between all annotated degree and essentiality when controlling for either intracomplex degree, intercomplex degree, or the size of the largest complex to which the protein belongs. Starred 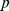-values indicate those with values 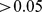. (**c**)**–**(**f**) The degree distribution of non-essential proteins is compared to that of essential proteins within complexes for (c) all annotated degree, (d) intracomplex degree, (e) intercomplex degree, and (f) complex size. In each box plot, the horizontal bar within a box corresponds to the median of the distribution; the two ends of the box indicate the first and third quartiles; and the small circles show outliers within the 2–98th percentile range. The significance of the difference between the two degree distributions is measured by the Wilcoxon rank sum test.

For each network, we derived a subnetwork where nodes represent proteins involved in any protein complex and edges represent interactions from our original interactions between these proteins. In the *Direct* network, 35.66% (or 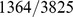) of interactions are intracomplex (Table S2). Repeating the analysis we performed for intraprocess vs. interprocess interactions, we found that intracomplex physical interactions are more correlated with protein essentiality than all annotated physical interactions (Figures 2 (a), S4 (a) and S5 (a)).

It has been previously observed that there is a strong correlation between complex size and essentiality [19], and argued that essential complexes tend to be large, and proteins within them tend to have more interactions, and this is a driving force in the relationship between essentiality and interaction degree. In our dataset, there is a clear positive correlation between complex size and the fraction of essential proteins within the complex (SRCC: 0.24, 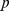-value: 2e-6). Moreover, there is a strong correlation between protein essentiality and the size of the largest complex to which it belongs, with SRCCs of 0.25, 0.24 and 0.24 for *Direct*, *Pull-down* and *Full* networks, respectively (Figures 2 (b), S4 (b) and S5 (b) ). We found, however, this relationship is not as strong as that between essentiality and intracomplex degree in our networks (black vs. green curve in Figures 2 (a), S4 (a) and S5 (a)).

We also computed partial correlations between essentiality and all annotated interactions, when controlling for intracomplex degree, intercomplex degree, or complex size. We found that when controlling for intracomplex degree, the SRCC between total degree and essentiality notably diminished (from 0.17, 0.32 and 0.32 to −0.01, 0.13 and 0.14 for the *Direct*, *Pull-down* and *Full* networks, respectively), whereas when controlling for intercomplex degree or complex size, the SRCC was not as greatly diminished (Figures 2 (b), S4 (b) and S5 (b)). Further, the difference in degree distribution between essential and non-essential proteins (Figuress 2, S4 and S5 (c)–(f)) is most significant when considering intracomplex degree and least significant when considering intercomplex degree. We note that there is a correlation between a protein's intracomplex degree and the size of the complex to which it belongs (SRCC: 0.3790, 0.7319 and 0.7809 for the *Direct*, *Pull-down* and *Full* networks, respectively); the much stronger correlations for the *Pull-down* and *Full* networks as compared to the *Direct* network are expected as the former two networks include many indirect (i.e., co-complex) interactions.

Thus far, we have found a stronger correlation between essentiality and intramodular degree than between essentiality and all annotated degree when we focus on either biological process or protein complex derived modules. Instead of using biological process or protein complex annotations to categorize interactions as either intramodular or intermodular, we next considered modules derived from network clustering approaches. In particular, we applied the state-of-the-art SPICi network clustering algorithm [31], and categorized interactions within clusters as intramodular and interactions between clusters as intermodular. We note that clusters are uncovered in a purely topological manner and may correspond to either protein complexes or functional modules. On the *Direct*, *Pull-down* and *Full* networks, essentiality is more correlated with intramodular interaction degree, defined via network clustering, than it is with either intermodular or total degree (Figure S7).

**Figure 3:**
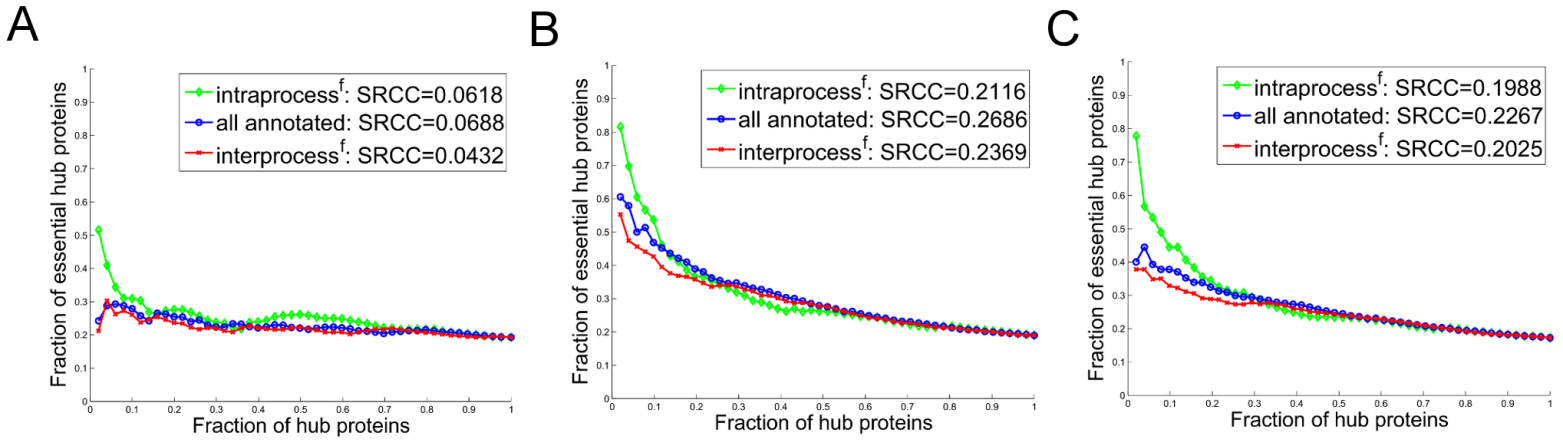
In all three networks ((a) *Direct*, (b) *Pull-down*, and (c) *Full* network), for a set of biological processes filtered to remove the effects of complexes, the intraprocess interaction degree is not more correlated with protein essentiality than the overall interaction degree. The fraction of essential proteins among hub proteins as a function of an increasing number of proteins considered as hub proteins; this is done by adding proteins in groups of 50 in a non-increasing order of the interaction degree. The relationship between protein essentiality and interaction degree is shown for intraprocess interactions (green), all annotated interactions (blue) and interprocess interactions (red). Correlations measured by SRCCs between essentiality and each type of interaction degree are also given.

What happens if we consider intraprocess interactions when excluding those that are intracomplex? That is, some biological processes may consist of a single protein complex or several protein complexes; in these cases some of the observed intraprocess interactions are more specifically intracomplex interactions within complexes that take part in the process. To focus on interactions that are not intracomplex, we filtered biological processes to remove these interactions (see Materials and Methods for more details). Among the proteins that are annotated with any filtered biological process, 16.52% (or 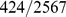) proteins are essential, which is slightly less than that when considering all proteins in the genome. In a subnetwork for the set of filtered biological processes from each of three interaction networks, there is a weaker correlation between interaction degree and essentiality as compared to the correlation for complexes, and the intraprocess degree is not more correlated with essentiality than all annotated degree (Figure 3 (a),(b),(c)). The correlations are especially weak in the *Direct* network. Moreover, in the *Pull-down* and *Full* networks, the correlation between essentiality and interprocess filtered interaction degree is somewhat higher than that between essentiality and intraprocess filtered degree.

### Essential proteins are more central within essential protein complexes

Having shown in a global analysis of proteins within complexes that essential proteins tend to have more intracomplex interactions than non-essential proteins, we next considered a per-complex analysis. We hypothesized that, for each essential protein complex, its essential proteins should be more central or have a higher intracomplex degree than its non-essential proteins. We tested this hypothesis for a subset of protein complexes with enough member proteins and intracomplex interactions. In particular, we included a complex in our test if it has at least two essential proteins and at least two non-essential proteins, each of which has intracomplex interactions. Table 1 shows that for a large fraction of complexes, essential proteins tend to have a higher average intracomplex degree than non-essential proteins. In particular, in the *Direct* network, for more than 76% of complexes, essential proteins have higher average intracomplex degree (empirical 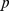-value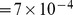). In the *Pull-down* or the *Full* network, the fraction of complexes with a higher average degree for essential proteins is lower than in the *Direct* network (58.8% and 61.5%, respectively); this is as expected since these networks include indirect intracomplex interactions. In fact, in the *Pull-down* and the *Full* networks, there are seven “clique” complexes in which every protein has an intracomplex interactions with all other proteins within the complex, whereas there are no such complexes in the *Direct* network. Without these clique complexes, the percent of complexes with higher average intracomplex degree for essential proteins goes up to 68.2% and 71.1% for the *Pull-down* and the *Full* networks, respectively.

**Table 1:** Within each essential protein complex, essential proteins tend to have a higher average intracomplex degree.

	Num ComplexesTested	Num Complexes with HigherAvg Essential Degree	Empiricalp-value
***Direct***	38	29 (76.32%)	7e-4
***Pull-down***	51	30 (58.82%)	3e-3
***Full***	52	32 (61.54%)	1e-3

**Num Complexes Tested** gives the number of complexes considered in each of the three networks; each such complex was required to have at least two essential proteins and at least two non-essential proteins, each with intracomplex interactions. **Num Complexes with Higher Avg Essential Degree** gives the number of complexes among the tested complexes where the essential proteins have higher intracomplex degree on average than the non-essential proteins. To determine whether this number is significant, we randomly permuted essentiality assignments and computed the number of complexes with higher average intracomplex degrees for essential proteins. **Empirical** 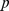 **-value** gives the fraction of random permutations where the number of such complexes is greater than or equal to the actual number, computed over 10,000 permutations.

By considering each complex individually, this analysis better handles proteins involved in multiple complexes. Although we removed highly overlapping complexes (see Materials and Methods), 14% (or 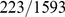) of proteins belong to two or more complexes. Moreover, these proteins tend to be essential; among proteins in more than one complex, 53.81% (or 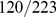) are essential (as opposed to 37.54% for all proteins within complexes). Thus, it is possible that one reason that essential proteins tend to have a higher intracomplex degree (Figure 2) is because essentiality is enriched in proteins belonging to multiple complexes, and the intracomplex degree of an essential protein is computed over the complexes to which it belongs to; however, looking at one complex at a time should alleviate this problem.

**Figure 4:**
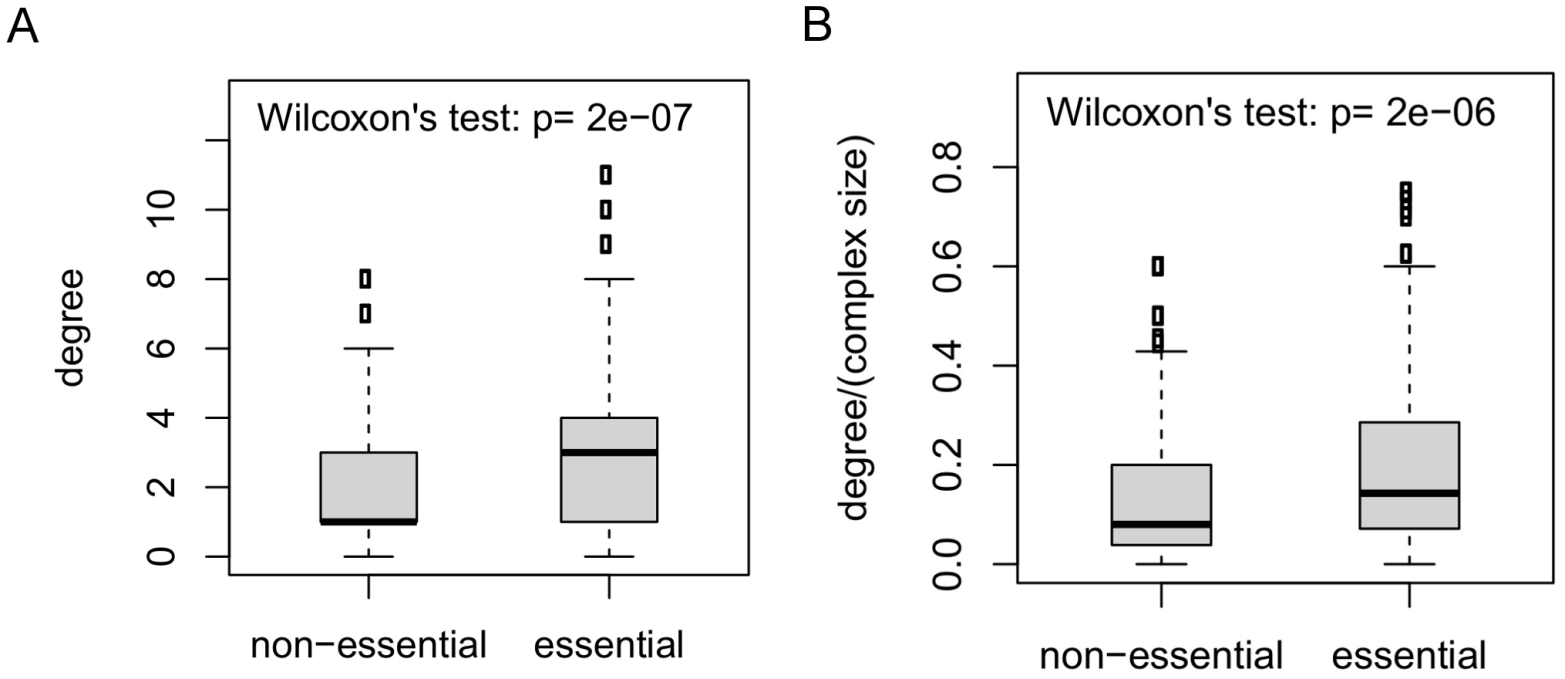
Within essential complexes, essential proteins tend to have a higher intracomplex degree in the *Direct* network than non-essential proteins. (**a**) The intracomplex degree or (**b**) the normalized intracomplex degree of essential proteins is significantly larger than that of non-essential proteins. Only protein complexes that have at least two essential proteins and at least two non-essential proteins, each with intracomplex interactions, are considered. Outliers within the 2–98th percentiles are shown. The significance of the difference between the two degree distributions is determined by the Wilcoxon rank sum test.

As another way of addressing the possible bias due to proteins participating in multiple complexes, for each protein, we computed the intracomplex degree using only interactions within the largest complex to which it belongs. Next, we compared all proteins within complexes, and found that there is a significant difference in degree distribution between essential and non-essential proteins (Figure 4 (a)), and this is also true in the other two networks (Figures S8 (a) and S9 (a)). Since there is a correlation between complex size and the fraction of essential proteins within the complex [19], and complex size is also correlated with the intracomplex degree of its member proteins, it is possible that the observed relationship between intracomplex degree and essentiality is due to the correlation between the complex size and essentiality. To address this, we next normalized interaction degree by complex size; that is, the normalized intracomplex degree of a protein is computed as the number of intracomplex interactions divided by the complex size. We found that the normalized degree of essential proteins tends to be significantly greater than that of non-essential proteins (Figures 4 (b), S8 (b) and S9 (b)).

### Essential complexes and processes tend to have higher cross-talk degree in a module-level network

As we have just shown, essential proteins tend to have more intramodular interactions, and for complexes with essential proteins, its essential proteins tend to have more intracomplex interactions than its non-essential proteins. In contrast, the intermodular interaction degree of a protein has a weaker relationship with its essentiality. Nevertheless, as noted earlier, there are a significant number of intermodular physical interactions (see Table S2), and presumably these physical interactions connecting different functional modules in the network are important for the module to accomplish a task.

We hypothesized that the essentiality of a protein complex or functional module may be related to its topological prominence within a module-level network. To test this, we built a “module network” where nodes are modules and edges are between modules that have an enriched number of intermodular cross-talk interactions. In particular, we constructed a module network for either protein complexes or filtered biological processes from each physical interaction network by computing the number of physical interactions between two modules and comparing this to the average number found in randomized networks (see Materials and Methods). For each network, we give the number of cross-talks uncovered using modules derived either from protein complexes or filtered biological processes in Tables 2 and 3, respectively. We note that the number of cross-talks for processes is much higher than that for complexes because a relatively higher number of interactions for processes are intermodular rather than intramodular (86.98% vs. 64.34%, Table S2).

**Table 2:** Module-level networks for protein complexes.

Network	Num Cross-talks	Num Modules	Fraction of Essential Modules
***Direct***	194	143	0.68
***Pull-down***	535	242	0.60
***Full***	727	279	0.56

A module-level network was built for protein complexes using each of the three networks. **Num Cross-talks** gives the number of inferred cross-talks. **Num Modules** gives the number of modules with at least one inferred cross-talk. **Fraction of Essential Modules** gives the fraction of modules having at least one essential protein, amongst modules with at least one cross-talk.

**Table 3:** Module-level networks for filtered biological processes.

Network	Num Cross-talks	Num Modules	Fraction of Essential Modules
***Direct***	1149	307	0.79
***Pull-down***	1409	321	0.77
***Full***	2306	371	0.74

A module-level network was built for filtered biological processes using each of the three networks. **Num Cross-talks** gives the number of inferred cross-talks. **Num Modules** gives the number of modules with at least one inferred cross-talk. **Fraction of Essential Modules** gives the fraction of modules having at least one essential protein, amongst modules with at least one cross-talk.

**Figure 5:**
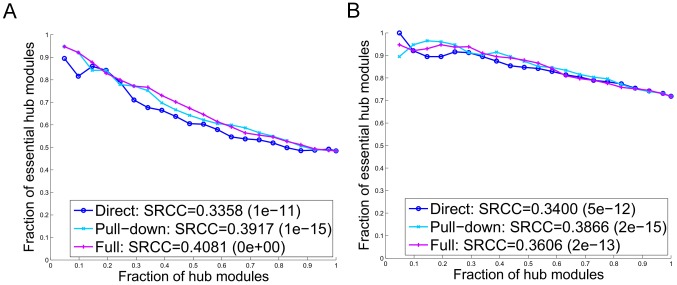
Cross-talk degree in a module-level network is correlated with module essentiality. For either (a) protein complexes or (b) filtered biological processes, the fraction of modules containing at least one essential protein among “hub modules” tends to decrease in each network as more modules are considered hubs. For the data shown, modules are added in groups of 20 in a non-increasing order of cross-talk degree in the *Direct* (blue), *Pull-down* (cyan) and *Full* (purple) networks. Correlations computed using the SRCC are shown for each network between the binary essentiality of a module and its inferred cross-talk degree. The binary essentiality for a module is 1 if the module has at least one essential protein, and 0 otherwise.

**Figure 6:**
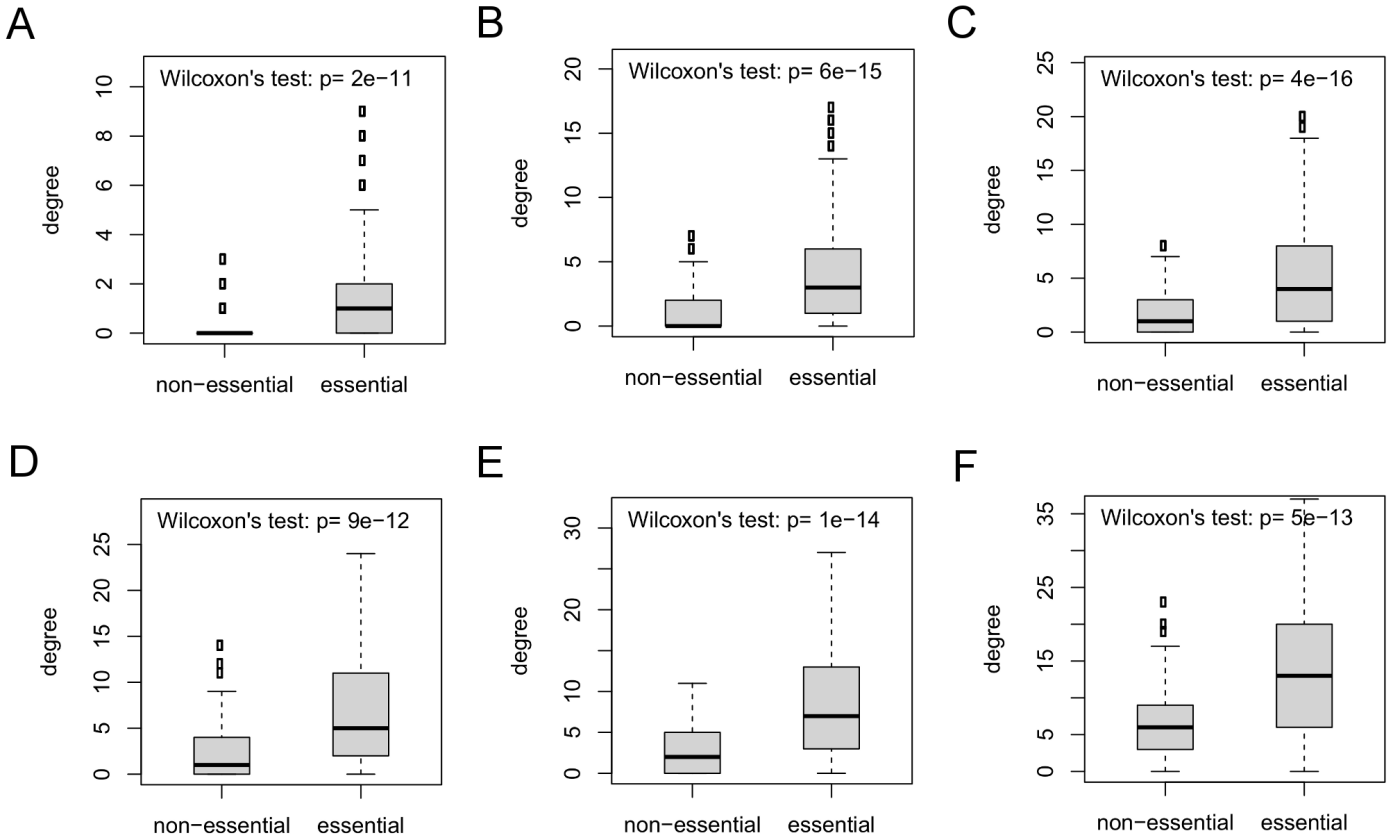
The cross-talk degree distribution of non-essential modules is compared to that of essential modules in the (a) *Direct*, (b) *Pull-down* and (c) *Full* networks for protein complexes and (d) *Direct*, (e) *Pull-down* and (f) *Full* networks for filtered biological processes. In each case, modules with essential proteins have significantly higher cross-talk degree than modules without essential proteins, as determined by the Wilcoxon rank sum test.

For modules, defined by either complexes or filtered biological processes, as we decrease the threshold for the number of cross-talks required for a module to be a considered a hub module, we find that the fraction of modules that contain an essential protein tends to decline (Figure 5). Further, there is a significant positive correlation between whether a module contains an essential protein and its cross-talk degree, with SRCCs on the three networks 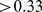 when considering complexes and 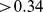 when considering filtered biological processes. Since modules that have more proteins may also have larger cross-talk degree, we also computed the partial correlation between cross-talk degree and module essentiality when controlling for the number of proteins in the module (Table S3); this varies in the three networks from 0.22–0.29 when considering complexes and 0.23–0.28 when considering filtered biological processes. Further, we found a significantly positive correlation between the normalized cross-talk degree of a module, defined as the cross-talk degree divided by module size, and module essentiality (Table S3). We also compared the cross-talk degree distribution between essential and non-essential modules using the Wilcoxon rank sum test. In our three networks, whether considering protein complexes or biological processes, the essential modules have significantly higher cross-talk degree than non-essential modules (Figure 6). Finally, since modules with a larger number of proteins have a greater chance of containing an essential protein, we also considered the fraction of proteins within a module that are essential. We found that the cross-talk degree of a module is positively correlated with the fraction of proteins within a module that are essential (Table S4), though these values are not as high as for binary essentiality (SRCCs on the three networks 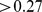 for complexes and 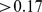 for filtered biological processes).

**Figure 7:**
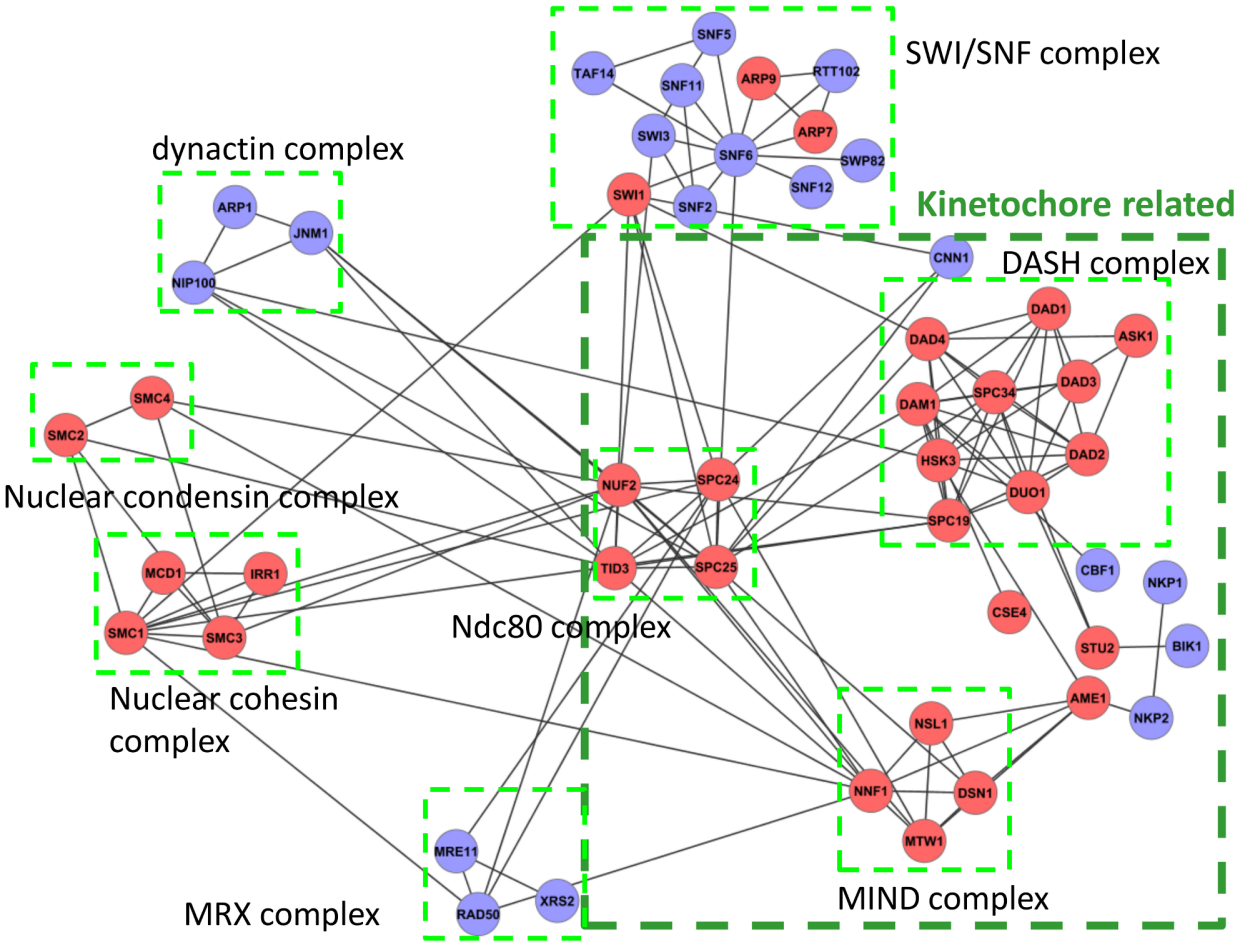
Cross-talk network centered on the Ndc80 complex. Cross-talk analysis on the *Direct* network uncovers seven cross-talks between the Ndc80 complex and other annotated complexes. The Ndc80 complex is part of the kinetochore, and two of the cross-talks are with other subcomplexes within the kinetochore (shown within the dark green box), with the remaining cross-talks with other non-kinetochore complexes. An individual proteins is colored red if it is essential, and blue otherwise.

We observed that many cross-talks occur between functional modules that are functionally related (i.e., they both take part in a more general, shared biological process). These types of cross-talks can be interpreted as intraprocess interactions at a broader level of functional similarity. As one example, the Ndc80p complex has a high cross-talk degree in all networks studied. In the *Direct* network, we uncover seven cross-talks (Figure 7). Ndc80p is a component of the kinetochore, which is central to chromosome segregation and couples chromosomes to microtubule polymers. Two of the uncovered cross-talks are with the DASH and MIND complexes, both of which are also kinetochore associated; these cross-talks can be thus be interpreted as “intramodular” interactions at a higher level of organization. On the other hand, Ndc80 also has cross-talks with other complexes that take part in a range of distinct biological processes, including the nucleosome remodeling complex SWI/SNF, the dynactin microtubule associated complex, the MRX complex involved in DNA damage repair, the nuclear condensin complex and the nuclear cohesion complex.

To see if essential functional modules have many cross-talks with functional modules representing truly different biological processes, we considered a set of expert-selected GO BP terms in yeast [32], and focused on those that annotate at most 500 proteins. We considered a functional module to be annotated with one of these terms if 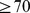
% of its proteins are annotated with it. We next ignored cross-talks between two functional modules if they are both annotated with a shared term; even in this case, we found that essential functional modules are still correlated with cross-talk degree (Table S5). Thus, a range of analyses reveals that there is a relationship between the topological importance of a functional module and its tendency to contain essential proteins.

### Analysis on high-throughput networks

Because low-throughput studies may be biased towards studying essential proteins, essential proteins may appear to have more interactions in existing network databases. Further, high-throughput studies may themselves utilize a select set of “bait” proteins that may bias the degree distribution of interaction networks. To address these potential concerns, we performed several additional network analyses.

First, we repeated our analysis on the *Y2H-union* network [21] and the more recently built *BinaryHQHT* network [33], both derived from high-throughput yeast two-hybrid data. In these networks, interactions found in experiments involving a few bait proteins were removed, and only high-quality yeast-two hybrid interactions (found in several experiments) were retained. The networks have notably smaller size when focusing on annotated interactions (Tables S9, S10 and S11); nevertheless, as we outline below, repeating the analysis yields similar results as for the *Direct* network.

In the *Y2H-union* and *BinaryHQHT* networks, the intraprocess interaction degree of a protein has a weak but statistically significant correlation with its essentiality while the overall degree of a protein is not correlated with essentiality in the *Y2H-union* network and is only weakly correlated with essentiality in the *BinaryHQHT* network (Figures S10 and S11, (a) and (b)). That is, protein essentiality is reflected in intraprocess degree in these networks, not overall degree. Further, the intraprocess degree is found to be significantly higher for essential proteins than non-essential proteins (Figure S10 (d) and S11 (d)), but this is not true for overall degree and for interprocess degree (Figures S10 and S11, (c) and (e)). For 84.62% and 78.57% of essential protein complexes in the *Y2H-union* and *BinaryHQHT* networks, essential proteins tend to have a higher average intracomplex degree than non-essential proteins (Table S12), and essential proteins have higher intracomplex degree and normalized intracomplex degree than non-essential proteins (Figures S12 and S13). Next, in our module-level analysis, we find that the cross-talk degree in a complex-level network is significantly correlated with complex essentiality in the *BinaryHQHT* network (Figure S14(a)), and the cross-talk degree in a process-level network is significantly correlated with process essentiality on both networks (Figures S14 (b) and S15 (c) and (d)). For the *Y2H-union* network, only four complexes are found to have cross-talks, and the relationship between cross-talk degree and complex essentiality is weak (Figures S14 (a) and S15 (a)); this may be due to the small number of intercomplex interactions in this network (Table S10).

In our second analysis, we removed interactions uncovered in low-throughput experiments (where less than 50 interactions were determined) from the *Pull-down* network, and restricted our analysis to the interaction properties of proteins labelled as bait proteins. Bait proteins have a higher fraction of essential proteins in this network (Table S14). When considering just bait proteins, we find stronger relationships between intraprocess degree and essentiality than between overall interaction degree and essentiality (Figure S16 (a) and (b)). Moreover, the relationship between overall interaction degree and essentiality is no longer significant when controlling for intraprocess degree (Figure S16 (b)). Further, the intraprocess degree of bait proteins is found to be higher for essential proteins than non-essential proteins (Figure S16), and within complexes with both essential bait and non-essential bait proteins, essential proteins have higher intracomplex degree (Figure S17).

Overall, the relationships between intraprocess degree and protein essentiality, degree within complexes and essentiality, and module essentiality and cross-talk degree are largely recapitulated in the smaller networks, *Y2H-union* and *BinaryHQHT* networks, where low-throughput experiments and experiments biased towards essential proteins are specifically excluded. Further, comparisons between bait proteins, which may be enriched in interactions, also confirms a relationship between the number of (intraprocess) interactions a protein has and whether it is essential.

## Discussion

A long line of previous research has studied the relationship between network topology and protein essentiality. Recent work has argued that hubs take part in densely connected essential complexes and processes [18], and these essential complexes tend to be large [19]. That is, it has been argued that essentiality is a modular property, and essential proteins within essential modules tend to have many interactions as these modules tend to be large. Our initial analysis, revealing that a protein's intramodular interaction degree is more predictive of essentiality than its overall degree, largely supports this argument. We also found that if we focus on proteins that do not belong in complexes, the intraprocess interaction degree does not correlate with essentiality any better than overall interaction degree; this suggests that the observed network modularity of essential proteins is largely due to complexes, and is not a more general feature of biological processes.

The observed positive correlation between protein essentiality and intramodular degree cannot be attributed only to module-level complex essentiality. In particular, within essential protein complexes, we found that their essential proteins tend to have higher intracomplex degrees than their non-essential counterparts. That is, within essential complexes, the topological prominence of its constitutent proteins is related to essentiality; this may be due to the importance of these proteins in maintaining the structural integrity of these complexes. This view is consistent with the relative enrichment of essentiality amongst proteins with many structural interfaces as opposed to just one or two structural interfaces [34].

While we found that intermodular interactions were less important than intramodular interactions in explaining protein essentiality, we also observed a significant number of intermodular interactions in physical interaction networks. We considered these interactions at a modular level, and demonstrated that essential functional modules tend to have more cross-talks with other functional modules. That is, our analysis showed that there is correlation between network topology and essentiality both at the protein level as well as at the modular level. Further, we observed that functionally related modules are likely to interconnect to each other, thereby revealing the hierarchical structure of physical interaction networks.

Overall, our work has advanced our understanding of the relationship between essentiality and network topology. We have shown the importance of intramodular interactions, especially intracomplex interactions, and demonstrated that essential modules tend to have a higher cross-talk degree than non-essential modules. These findings are likely to yield improvements in our ability to predict protein essentiality. Indeed, integrative machine learning approaches that use a range of network and sequence features have been previously applied to predict protein essentiality (e.g., see [35]–[41]); based on our work, information about functional modules and protein complexes, especially with respect to intramodular and cross-talk degree, should also be incorporated within these frameworks.

In the future, it would be interesting to characterize the network properties of essential proteins that are not central in protein physical interaction networks. Based on our current findings, we can speculate that some of these proteins are important for the functioning of specific essential modules, and this may be reflected in their interactions with other proteins within their modules, but these relationships may be better represented via other types of interactions (e.g., regulatory, metabolic or genetic). Our framework for incorporating functional information into network analysis is likely to be useful in establishing whether or not this is the case. Finally, while we have performed our analysis on *S. cerevisiae*, our approach can be applied to study essential proteins in other well-annotated organisms with large-scale interaction networks and genome-scale gene deletion or disruption data.

## Materials and Methods

### Physical interaction datasets

We performed our analysis on five physical interaction datasets. For our first network, physical interactions were gathered from BioGRID [26], release 3.1.78, using all evidence codes indicative of physical interactions except “Affinity Capture-RNA” and “Protein-RNA.” For the early yeast two-hybrid paper of Ito *et al.* [42], we only included the core data. To remove artifacts due to “sticky proteins” in certain experiments, if a protein has more than 30 interactions from a single experimental data source, we removed these interactions. For our second network, we extracted direct physical interactions from the initial network by utilizing only interactions that were determined from one of the following experimental systems: Biochemical activity, Co-crystal structure, Far western, FRET, Protein-peptide, Reconstituted complex, and Two-hybrid. For our third network, we extracted from the initial network those interactions that were determined either by Affinity capture-Western or Affinity capture-MS. We refer to these three networks as *Full*, *Direct* and *Pull-down*, respectively, and their sizes are given in Table S1.

We also considered two additional networks, comprised of interactions that were not determined in small-scale experimental assays; in this manner, we attempt to minimize the effect of study bias. The first of these networks, which we refer to as *Y2H-union*, was built in an earlier study [21]; it included only interactions determined in large-scale high-quality yeast two-hybrid studies, and excluded an experiment using a specific set of “bait” proteins that was enriched in essential proteins [43]. We next used the more recently built high-throughput yeast two-hybrid network of [33], which we refer to as *BinaryHQHT*. Finally, we built a high-throughput network from our *Pull-down* network by keeping only those interactions that were found in experiments uncovering at least 50 interactions and for which there were more than 10 “bait” proteins. We refer to this second network as the *Pull-down^f^* network, and use it to compare the network properties of bait proteins with respect to each other.

### Protein complexes and biological processes

We used the set of 430 protein complexes compiled in [27], which includes the SGD Macromolecular Complex GO standard [44], the CYC2008 protein complex catalog [45] and a set of manually curated complexes. From this initial set, we removed highly overlapping complexes as follows. First, if the proteins comprising one complex are a subset of the proteins comprising another complex, the smaller complex is removed. Next, for any two complexes, if the Jaccard index of the proteins making them up (i.e., the number of overlapping proteins divided by the size of the union of the protein sets) is 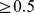, we removed the smaller complexes. Additionally, as in previous work [19], we removed the four complexes corresponding to the subunits of the ribosome, as they contain a large number of proteins; that is, these four complexes can disproportionately affect the per-protein analysis. After these filters, we were left with 390 complexes. (See the Supplement Figure S6, Text S1 for intraprocess and interprocess results including the four ribosomal complexes).

For our functional analysis, we worked with a subset of specific Gene Ontology (GO) Biological Process (BP) terms [25] that were derived from the entire GO (version 1.1.2130) as follows. First, we extracted 1418 BP terms, each of which annotates at least 5 yeast proteins and at most 50. Next, to hone in on the contribution of a specific biological process (as opposed to the effects arising from proteins that are annotated with that process but are within protein complexes), we pruned the set of proteins that are associated with these functional terms. More specifically, if the size of the intersection between a biological process and one of our original set of 430 protein complexes is 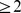, the proteins in the intersection were no longer associated with the process. If this left fewer than 2 proteins associated with the process, or with less than half the number of proteins that it is known to annotate, then this term was removed from consideration. Finally, highly overlapping processes were removed in the same manner as described above for complexes. This procedure resulted in 391 “filtered” processes, with 2567 proteins associated with at least one of these processes.

### Detecting cross-talk between complexes and processes

For a given network, we exhaustively determined whether pairs of functional modules are enriched in the number of interactions found between them [10]. We considered modules arising from complexes or processes in turn (i.e., functional modules consist of either proteins within the same complex, or that have a shared process annotation from the 391 filtered processes considered). We considered the proteins within the network that are associated with any of the modules that we are considering, as well as all the edges that correspond to intermodular interactions amongst these proteins. Next, for any two modules 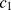 and 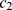 we counted the number of “cross-talk” interactions between the proteins comprising each of these modules. Note that interactions where either of the proteins is annotated with both 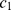 and 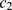 were not included as these are intramodular interactions. To determine whether the number of observed cross-talk interactions for this pair is more than would be expected by chance, we randomized the intermodular interactions within the network 100 times using stub-rewiring (as in [8]), thereby preserving degree distribution, module annotation, and the overall number of cross-talk interactions. Then, if 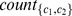 is the number of cross-talk interactions between 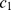 and 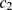 in the real network, and 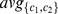 is the average number of corresponding cross-talk interactions in randomized networks, the odds-score of the module pair is defined as:
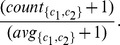



The addition of the pseudocount of 1 downweighs the contribution of very rare cross-talks that could otherwise obtain high scores simply due to very small (or zero) average counts in the randomized graphs. In order for a module pair to be considered a cross-talk, we required that there should be at least two independent (i.e., non-overlapping) cross-talk interactions, and that its odds-score should be at least 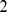. The observed relationship between module essentiality and cross-talk degree persists for a range of odds-scores (see Tables S6–S8).

### Semantic similarity

The semantic similarity between two GO terms within the same ontology is an estimate of the functional similarity between the terms. We use the semantic similarity measure introduced by [30]. In particular, let 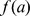 be the fraction of proteins in yeast annotated with term 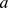 among the total number of proteins. Then 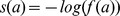 is a measure of how specific a term 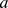 is. We compute the term semantic similarity of 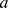 and 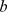, 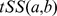 as 
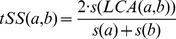
, where 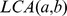 is a least common ancestor of 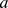 and 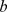 in the GO ontology. Note that if the LCA of two terms is a root term (e.g., GO:0008150 ‘biological process’), then 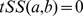. Moreover, if two terms are the same, then 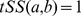.

This measure is naturally extended to functional relationships between proteins that have multiple annotations. For a protein 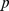, let 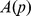 be the set of terms with which 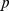 is annotated. If a term annotates 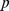, then all its parent terms are naturally included in 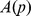. Then, between proteins 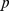 and 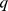, the protein semantic similarity (pSS) is defined as follows [7]:
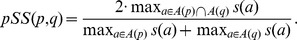



## Supporting Information

Figure S1
**The intraprocess interaction degree is more correlated with protein essentiality than the overall interaction degree for proteins in the** ***Pull-down*** **network, when interactions are categorized with specific BP terms, each of which annotates at most 50 proteins.** (**a**) The fraction of essential proteins among hub proteins as more proteins are considered hub proteins; proteins are added in groups of 50 in a non-increasing order of the interaction degree. This fraction is highest for intraprocess degree (green), followed by all annotated degree (blue) and then by interprocess degree (red). (**b**) The correlations measured by SRCCs between essentiality and either intraprocess, all annotated or interprocess degree. The SRCC is highest between essentiality and intraprocess degree. The partial correlation is also computed between all annotated degree and essentiality when controlling for either intraprocess or interprocess degree. Starred 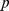-values indicate those with values 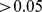. (**c**)**–**(**e**) The degree distribution of non-essential proteins is compared to that of essential proteins for (c) all annotated, (d) intraprocess, and (e) interprocess degree, respectively. In each box plot, the horizontal bar within a box corresponds to the median of the distribution; the two ends of the box indicate the first and third quartiles; and the small circles show outliers within the 2–98th percentile range. The significance of the difference between the two degree distributions is measured by the Wilcoxon rank sum test.(TIFF)

Figure S2
**The intraprocess interaction degree is more correlated with protein essentiality than the overall interaction degree for proteins in the** ***Full*** **network, when interactions are categorized with specific BP terms, each of which annotates at most 50 proteins.** (**a**) The fraction of essential proteins among hub proteins as more proteins are considered hub proteins; proteins are added in groups of 50 in a non-increasing order of the interaction degree. This fraction is highest for intraprocess degree (green), followed by all annotated degree (blue) and then by interprocess degree (red). (**b**) The correlations measured by SRCCs between essentiality and either intraprocess, all annotated or interprocess degree. The SRCC is highest between essentiality and intraprocess degree. The partial correlation is also computed between all annotated degree and essentiality when controlling for either intraprocess or interprocess degree. Starred 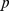-values indicate those with values 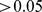. (**c**)**–**(**e**) The degree distribution of non-essential proteins is compared to that of essential proteins for (c) all annotated, (d) intraprocess, and (e) interprocess degree, respectively. In each box plot, the horizontal bar within a box corresponds to the median of the distribution; the two ends of the box indicate the first and third quartiles; and the small circles show outliers within the 2–98th percentile range. The significance of the difference between the two degree distributions is measured by the Wilcoxon rank sum test.(TIFF)

Figure S3 **In all three networks, the semantic similarity degree is more correlated with protein essentiality than the overall interaction degree.** (**a**)**–**(**c**) The fraction of essential proteins among hub proteins as more proteins are considered hub proteins for the *Direct*, *Pull-down* and *Full* networks; proteins are added in groups of 50 in a non-increasing order of the semantic similarity degree. For each network, the SRCC is computed between protein essentiality and either semantic similarity or all annotated degree; these values are boxed in each panel. (**d**)**–**(**f**) The semantic similarity weighted degree distribution of non-essential proteins is compared to that of essential proteins for the *Direct*, *Pull-down* and *Full* networks.
(TIFF)

Figure S4
**The intracomplex interaction degree is more correlated with protein essentiality than the overall interaction degree for proteins in the** ***Pull-down*** **network, when interactions are categorized using protein complexes.** (**a**) The fraction of essential proteins among hub proteins as more proteins are considered hub proteins; proteins are added in groups of 50 in a non-increasing order of the interaction degree or the size of the largest complex to which the protein belongs. The relationship between protein essentiality and interaction degree is shown for intracomplex interactions (green), all annotated interactions (blue) and intercomplex interactions (red). The relationship between protein essentiality and complex size is also shown (black). (**b**) The correlations measured by SRCCs between essentiality and either intracomplex degree, all annotated degree, intercomplex degree, or complex size. The SRCC is highest between essentiality and intracomplex degree. The partial correlation is also computed between all annotated degree and essentiality when controlling for either intracomplex degree, intercomplex degree, or the size of the largest complex to which the protein belongs. Starred 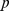-values indicate those with values 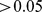. (**c**)**–**(**f**) The degree distribution of non-essential proteins is compared to that of essential proteins within complexes for (c) all annotated degree, (d) intracomplex degree, (e) intercomplex degree, and (f) complex size. In each box plot, the horizontal bar within a box corresponds to the median of the distribution; the two ends of the box indicate the first and third quartiles; and the small circles show outliers within the 2–98th percentile range. The significance of the difference between the two degree distributions is measured by the Wilcoxon rank sum test.(TIFF)

Figure S5
**The intracomplex interaction degree is more correlated with protein essentiality than the overall interaction degree for proteins in the** ***Full*** **network, when interactions are categorized using protein complexes.** (**a**) The fraction of essential proteins among hub proteins as more proteins are considered hub proteins; proteins are added in groups of 50 in a non-increasing order of the interaction degree or the size of the largest complex to which the protein belongs. The relationship between protein essentiality and interaction degree is shown for intracomplex interactions (green), all annotated interactions (blue) and intercomplex interactions (red). The relationship between protein essentiality and complex size is also shown (black). (**b**) The correlations measured by SRCCs between essentiality and either intracomplex degree, all annotated degree, intercomplex degree, or complex size. The SRCC is highest between essentiality and intracomplex degree. The partial correlation is also computed between all annotated degree and essentiality when controlling for either intracomplex degree, intercomplex degree, or the size of the largest complex to which the protein belongs. Starred 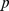-values indicate those with values 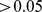. (**c**)**–**(**f**) The degree distribution of non-essential proteins is compared to that of essential proteins within complexes for (c) all annotated degree, (d) intracomplex degree, (e) intercomplex degree, and (f) complex size. In each box plot, the horizontal bar within a box corresponds to the median of the distribution; the two ends of the box indicate the first and third quartiles; and the small circles show outliers within the 2–98th percentile range. The significance of the difference between the two degree distributions is measured by the Wilcoxon rank sum test.(TIFF)

Figure S6 **The correlations between interaction degree and essentiality for proteins in all complexes, including ribosomal complexes, for (a)** ***Direct*** **, (b)** ***Pull-down*** **and (c)** ***Full*** **networks.** Interactions are categorized using protein complexes including ribosomal complexes. The fraction of essential proteins among hub proteins as more proteins are considered hub proteins; proteins are added in groups of 50 in a non-increasing order of the interaction degree or the size of the largest complex to which the protein belongs. The relationship between protein essentiality and interaction degree is shown for intracomplex interactions (green), all annotated interactions (blue) and intercomplex interactions (red). The relationship between protein essentiality and complex size is also shown (black).
(TIFF)

Figure S7 **Essentiality is more correlated with intramodular interaction degree than it is with intermodular or total degree, when modules are determined in each network ((a)** ***Direct*** **, (b)** ***Pull-down*** **and (c)** ***Full*** **) via network clustering approaches.** To obtain clusters, we used the SPICi clustering algorithm, a local clustering approach, with a density threshold of 0.5 and a minimum increment ratio of 0.3. The fraction of essential proteins among hub proteins as more proteins are considered hub proteins; proteins are added in groups of 50 in a non-increasing order of the interaction degree. The relationship between protein essentiality and interaction degree is shown for intramodular interactions (green), total interactions (blue) and intermodular interactions (red).
(TIFF)

Figure S8 **Within essential complexes, essential proteins tend to have a higher intracomplex degree in the** ***Pull-down*** **network than non-essential proteins.** (**a**) The intracomplex degree or (**b**) the normalized intracomplex degree of essential proteins is significantly larger than that of non-essential proteins. Only protein complexes that have at least two essential proteins and at least two non-essential proteins, each with intracomplex interactions are considered. Outliers within the 2–98th percentiles are shown. The significance of the difference of the two degree distributions is determined by the Wilcoxon rank sum test.
(TIFF)

Figure S9 **Within essential complexes, essential proteins tend to have a higher intracomplex degree in the** ***Full*** **network than non-essential proteins.** (**a**) The intracomplex degree or (**b**) the normalized intracomplex degree of essential proteins is significantly larger than that of non-essential proteins. Only protein complexes that have at least two essential proteins and at least two non-essential proteins, each with intracomplex interactions are considered. Outliers within the 2–98th percentiles are shown. The significance of the difference of the two degree distributions is determined by the Wilcoxon rank sum test.
(TIFF)

Figure S10
**The intraprocess interaction degree is more correlated with protein essentiality than the overall interaction degree for proteins in the** ***Y2H-union*** **network, when interactions are categorized with specific GO BP terms, each of which annotates at most 50 proteins.** (**a**) The fraction of essential proteins among hub proteins as more proteins are considered hub proteins; proteins are added in groups of size 20 (or larger so as to put proteins with the same degree in the same group). This fraction is highest for intraprocess degree (green), followed by all annotated degree (blue) and then by interprocess degree (red). (**b**) The correlations measured by SRCCs between essentiality and either intraprocess, all annotated or interprocess degree. The SRCC is highest between essentiality and intraprocess degree. The partial correlation is also computed between all annotated degree and essentiality when controlling for either intraprocess or interprocess degree. Starred 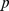-values indicate those with values 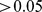. (**c**)**–**(**e**) The degree distribution of non-essential proteins is compared to that of essential proteins for (c) all annotated, (d) intraprocess, and (e) interprocess degree, respectively. In each box plot, the horizontal bar within a box corresponds to the median of the distribution; the two ends of the box indicate the first and third quartiles; and the small circles show outliers within the 2–98th percentile range. The significance of the difference between the two degree distributions is measured by the Wilcoxon rank sum test. For the *Y2H-union* network, essentiality and intraprocess degree have a small but statistically significant correlation. Essentiality is not significantly correlated with overall degree and interprocess degree. Further, essential proteins have higher average intraprocess degree than non-essential proteins in this network (panel (d)), while there is not a significant difference in all annotated degree or interprocess degree (panels (c) and (e)).(TIFF)

Figure S11
**The intraprocess interaction degree is more correlated with protein essentiality than the overall interaction degree for proteins in the** ***BinaryHQHT*** **network, when interactions are categorized with specific GO BP terms, each of which annotates at most 50 proteins.** (**a**) The fraction of essential proteins among hub proteins as more proteins are considered hub proteins; proteins are added in groups of size 20 (or larger so as to put proteins with the same degree in the same group). This fraction is highest for intraprocess degree (green), followed by all annotated degree (blue) and then by interprocess degree (red). (**b**) The correlations measured by SRCCs between essentiality and either intraprocess, all annotated or interprocess degree. The SRCC is highest between essentiality and intraprocess degree. The partial correlation is also computed between all annotated degree and essentiality when controlling for either intraprocess or interprocess degree. Starred 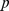-values indicate those with values 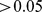. (**c**)**–**(**e**) The degree distribution of non-essential proteins is compared to that of essential proteins for (c) all annotated, (d) intraprocess, and (e) interprocess degree, respectively. In each box plot, the horizontal bar within a box corresponds to the median of the distribution; the two ends of the box indicate the first and third quartiles; and the small circles show outliers within the 2–98th percentile range. The significance of the difference between the two degree distributions is measured by the Wilcoxon rank sum test. For the *BinaryHQHT* network, essentiality and intraprocess degree have a small but statistically significant correlation. Essentiality has a smaller correlation with overall degree and is not correlated with interprocess degree. Further, essential proteins have higher average intraprocess degree than non-essential proteins in this network (panel (d)), while there is not a significant difference in interprocess degree (panel (e)).(TIFF)

Figure S12 **Essential proteins tend to have a higher intracomplex degree than non-essential proteins within protein complexes in the** ***Y2H-union*** **network.** (**a**) The intracomplex degree or (**b**) the normalized intracomplex degree of essential proteins is significantly greater than that of non-essential proteins. Only protein complexes that have at least two essential proteins and at least two non-essential proteins with intracomplex interactions are tested. Outliers within 2–98% are shown. The significance of the difference of the two degree distributions is determined by the Wilcoxon rank sum test.
(TIFF)

Figure S13 **Essential proteins tend to have a higher intracomplex degree than non-essential proteins within protein complexes in the** ***BinaryHQHT*** **network.** (**a**) The intracomplex degree or (**b**) the normalized intracomplex degree of essential proteins is significantly greater than that of non-essential proteins. Only protein complexes that have at least two essential proteins and at least two non-essential proteins with intracomplex interactions are tested. Outliers within 2–98% are shown. The significance of the difference of the two degree distributions is determined by the Wilcoxon rank sum test.
(TIFF)

Figure S14 **Cross-talk degree in a module-level network is correlated with module essentiality.** For either (a) protein complexes or (b) filtered biological processes, the fraction of modules containing at least one essential protein among “hub modules” tends to decrease in each network as more modules are considered hubs. For the data shown, modules are added in a non-increasing order of cross-talk degree in the *Y2H-union* (blue) and *BinaryHQHT* (cyan) networks. Correlations between the binary essentiality of a module and its inferred cross-talk degree are computed using the SRCC and are shown for each network. The binary essentiality for a module is 1 if the module has at least one essential protein, and 0 otherwise. For the *Y2H-union* module network comprised of complexes (panel (a)), the correlation is not significant as we uncover only four complexes with crosstalks in this small network (549 edges, see Table S10).
(TIFF)

Figure S15 **The cross-talk degree distribution of non-essential modules is compared to that of essential modules in the (a)** ***Y2H-union*** **and (b)** ***BinaryHQHT*** **networks for protein complexes and (c)** ***Y2H-union*** **and (d)** ***BinaryHQHT*** **networks for filtered biological processes.** For the *Y2H-union* and *BinaryHQHT* networks, modules (derived from biological processes) with essential proteins have significantly higher cross-talk degree than modules without essential proteins, as determined by the Wilcoxon rank sum test. For modules derived from complexes in the *Y2H-union* network, the differences between essential and non-essential modules are not significant, as there are only four modules for which we can uncover cross-talks.
(TIFF)

Figure S16
**The intraprocess interaction degree is more correlated with protein essentiality than the overall interaction degree for bait proteins in the** ***Pull-down^f^*** **network excluding small-scale experiments, when interactions are categorized with specific GO BP terms, each of which annotates at most 50 proteins.** All tests were done for only bait proteins in the *Pull-down^f^* network. (**a**) The fraction of essential proteins among hub proteins as more proteins are considered hub proteins; proteins are added in a non-increasing order of the interaction degree. This fraction is highest for intraprocess degree (green), followed by all annotated degree (blue) and then by interprocess degree (red). (**b**) The correlations measured by SRCCs between essentiality and either intraprocess, all annotated or interprocess degree. The SRCC is highest between essentiality and intraprocess degree. The partial correlation is also computed between all annotated degree and essentiality when controlling for either intraprocess or interprocess degree. Starred 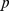-values indicate those with values 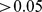. (**c**)**–**(**e**) The degree distribution of non-essential proteins is compared to that of essential proteins for (c) all annotated, (d) intraprocess, and (e) interprocess degree, respectively. In each box plot, the horizontal bar within a box corresponds to the median of the distribution; the two ends of the box indicate the first and third quartiles; and the small circles show outliers within the 2–98th percentile range. The significance of the difference between the two degree distributions is measured by the Wilcoxon rank sum test.(TIFF)

Figure S17 **Within essential complexes, essential bait proteins tend to have a higher intracomplex degree in the** ***Pull-down^f^*** **network than non-essential bait proteins.** (**a**) The intracomplex degree or (**b**) the normalized intracomplex degree of essential bait proteins is significantly larger than that of non-essential bait proteins. Only protein complexes that have at least two essential bait proteins and at least two non-essential bait proteins, each with intracomplex interactions, are considered. Outliers within the 2–98th percentiles are shown. The significance of the difference between the two degree distributions is determined by the Wilcoxon rank sum test.
(TIFF)

Table S1 **The number of proteins, the number of interactions and the fraction of essential proteins for each of the three physical interaction networks considered.**
(PDF)

Table S2 **A substantial fraction of physical interactions in the** ***Direct*** **network are intermodular.**
(PDF)

Table S3 **Correlation between cross-talk (CT) degree and binary module essentiality.**
(PDF)

Table S4 **Correlation between cross-talk (CT) degree and the fraction of essential proteins in the module.**
(PDF)

Table S5 **Correlation between cross-talk (CT) degree and binary module essentiality after removing functionally similar cross-talks.**
(PDF)

Table S6 **The significant correlation between cross-talk degree and binary module essentiality persists for a range of odd-scores in the** ***Direct*** **network.**
(PDF)

Table S7 **The significant correlation between cross-talk degree and binary module essentiality persists for a range of odd-scores in the** ***Pull-down*** **network.**
(PDF)

Table S8 **The significant correlation between cross-talk degree and binary module essentiality persists for a range of odd-scores in the** ***Full*** **network.**
(PDF)

Table S9 **The number of proteins, the number of interactions and the fraction of essential proteins for the** ***Y2H-union*** **and** ***BinaryHQHT*** **physical interaction networks.**
(PDF)

Table S10 **A substantial fraction of physical interactions in the** ***Y2H-union*** **network are intermodular.**
(PDF)

Table S11 **A substantial fraction of physical interactions in the** ***BinaryHQHT*** **network are intermodular.**
(PDF)

Table S12 **Within each essential protein complex, essential proteins tend to have a higher average intracomplex degree in** ***Y2H-union*** **and** ***BinaryHQHT*** **networks.**
(PDF)

Table S13 **Module-level networks for** ***Y2H-union*** **and** ***BinaryHQHT*** **networks.**
(PDF)

Table S14 **Numbers of bait proteins in the** ***Pull-down^f^*** **network.**
(PDF)

Table S15 **Within each essential protein complex, essential bait proteins tend to have a higher average intracomplex degree than non-essential bait proteins in the** ***Pull-down^f^*** **network.**
(PDF)

Text S1 **Analysis on the correlations between interaction degree and essentiality for proteins in all complexes, including ribosomal complexes, for all three networks.**
(PDF)
