# Supplementary figures and images for: From Hub Proteins to Hub Modules: The Relationship Between Essentiality and Centrality in the Yeast Interactome at Different Scales of Organization
Source: PLoS Comput Biol. 2013 Feb 21;9(2):e1002910. doi: 10.1371/journal.pcbi.1002910 (PMC3578755; doi:10.1371/journal.pcbi.1002910)

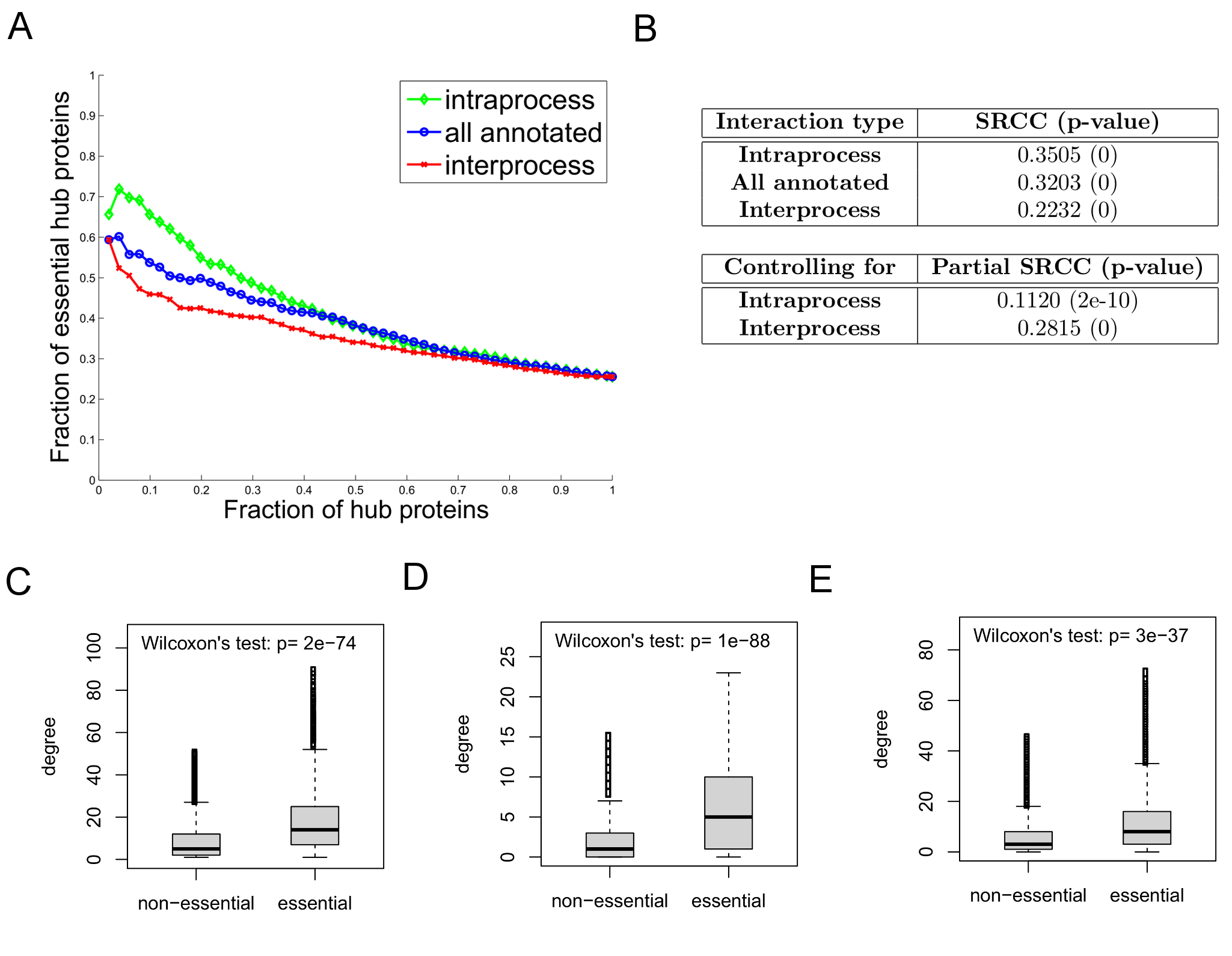

Supplement: Figure S1 — The intraprocess interaction degree is more correlated with protein essentiality than the overall interaction degree for proteins in the Pull-down network, when interactions are categorized with specific BP terms, each of which annotates at most 50 proteins. (a) The fraction of essential proteins among hub proteins as more proteins are considered hub proteins; proteins are added in groups of 50 in a non-increasing order of the interaction degree. This fraction is highest for intraprocess degree (green), followed by all annotated degree (blue) and then by interprocess degree (red). (b) The correlations measured by SRCCs between essentiality and either intraprocess, all annotated or interprocess degree. The SRCC is highest between essentiality and intraprocess degree. The partial correlation is also computed between all annotated degree and essentiality when controlling for either intraprocess or interprocess degree. Starred -values indicate those with values . (c)–(e) The degree distribution of non-essential proteins is compared to that of essential proteins for (c) all annotated, (d) intraprocess, and (e) interprocess degree, respectively. In each box plot, the horizontal bar within a box corresponds to the median of the distribution; the two ends of the box indicate the first and third quartiles; and the small circles show outliers within the 2–98th percentile range. The significance of the difference between the two degree distributions is measured by the Wilcoxon rank sum test. (TIFF) [file pcbi.1002910.s001.tiff]

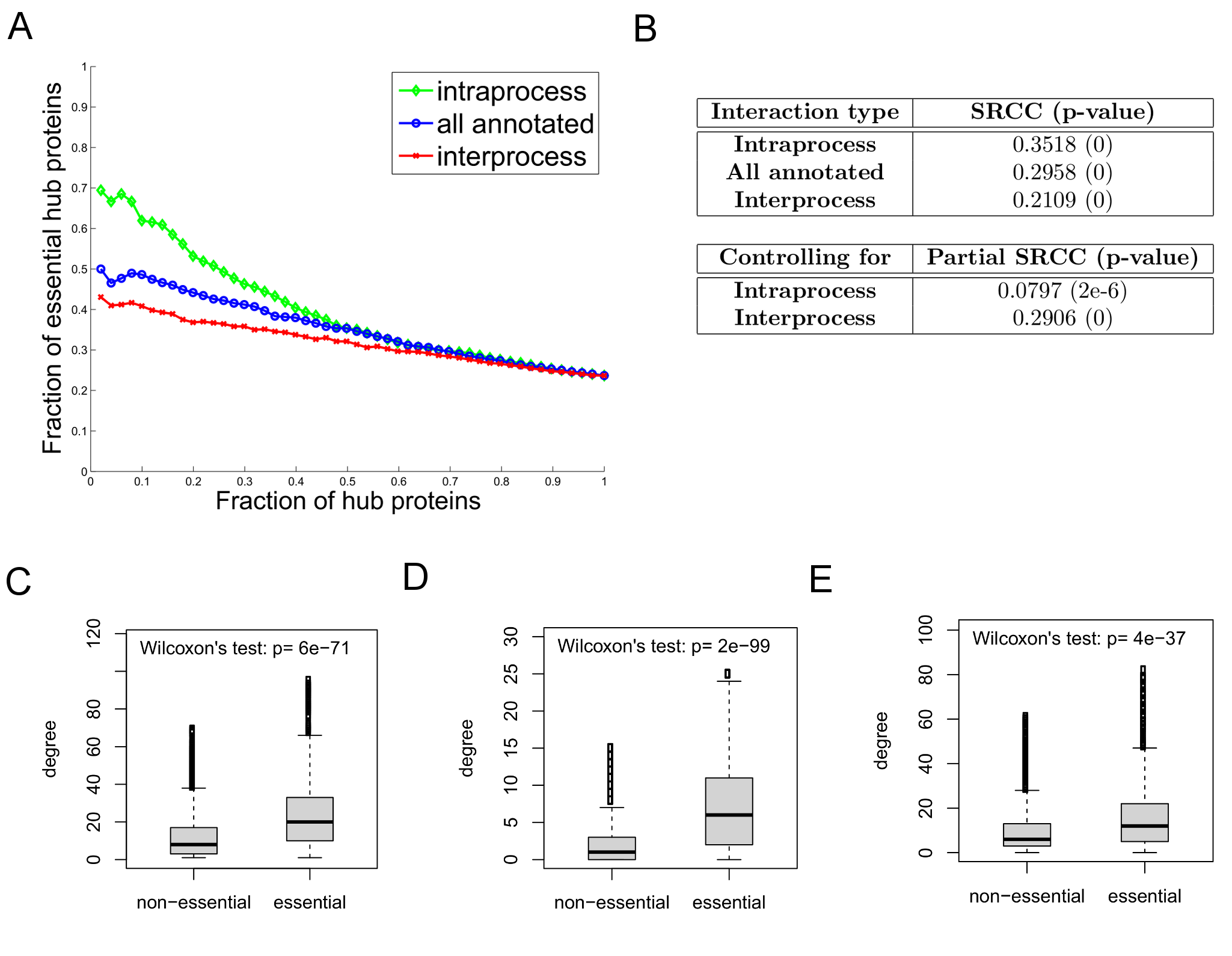

Supplement: Figure S2 — The intraprocess interaction degree is more correlated with protein essentiality than the overall interaction degree for proteins in the Full network, when interactions are categorized with specific BP terms, each of which annotates at most 50 proteins. (a) The fraction of essential proteins among hub proteins as more proteins are considered hub proteins; proteins are added in groups of 50 in a non-increasing order of the interaction degree. This fraction is highest for intraprocess degree (green), followed by all annotated degree (blue) and then by interprocess degree (red). (b) The correlations measured by SRCCs between essentiality and either intraprocess, all annotated or interprocess degree. The SRCC is highest between essentiality and intraprocess degree. The partial correlation is also computed between all annotated degree and essentiality when controlling for either intraprocess or interprocess degree. Starred -values indicate those with values . (c)–(e) The degree distribution of non-essential proteins is compared to that of essential proteins for (c) all annotated, (d) intraprocess, and (e) interprocess degree, respectively. In each box plot, the horizontal bar within a box corresponds to the median of the distribution; the two ends of the box indicate the first and third quartiles; and the small circles show outliers within the 2–98th percentile range. The significance of the difference between the two degree distributions is measured by the Wilcoxon rank sum test. (TIFF) [file pcbi.1002910.s002.tiff]

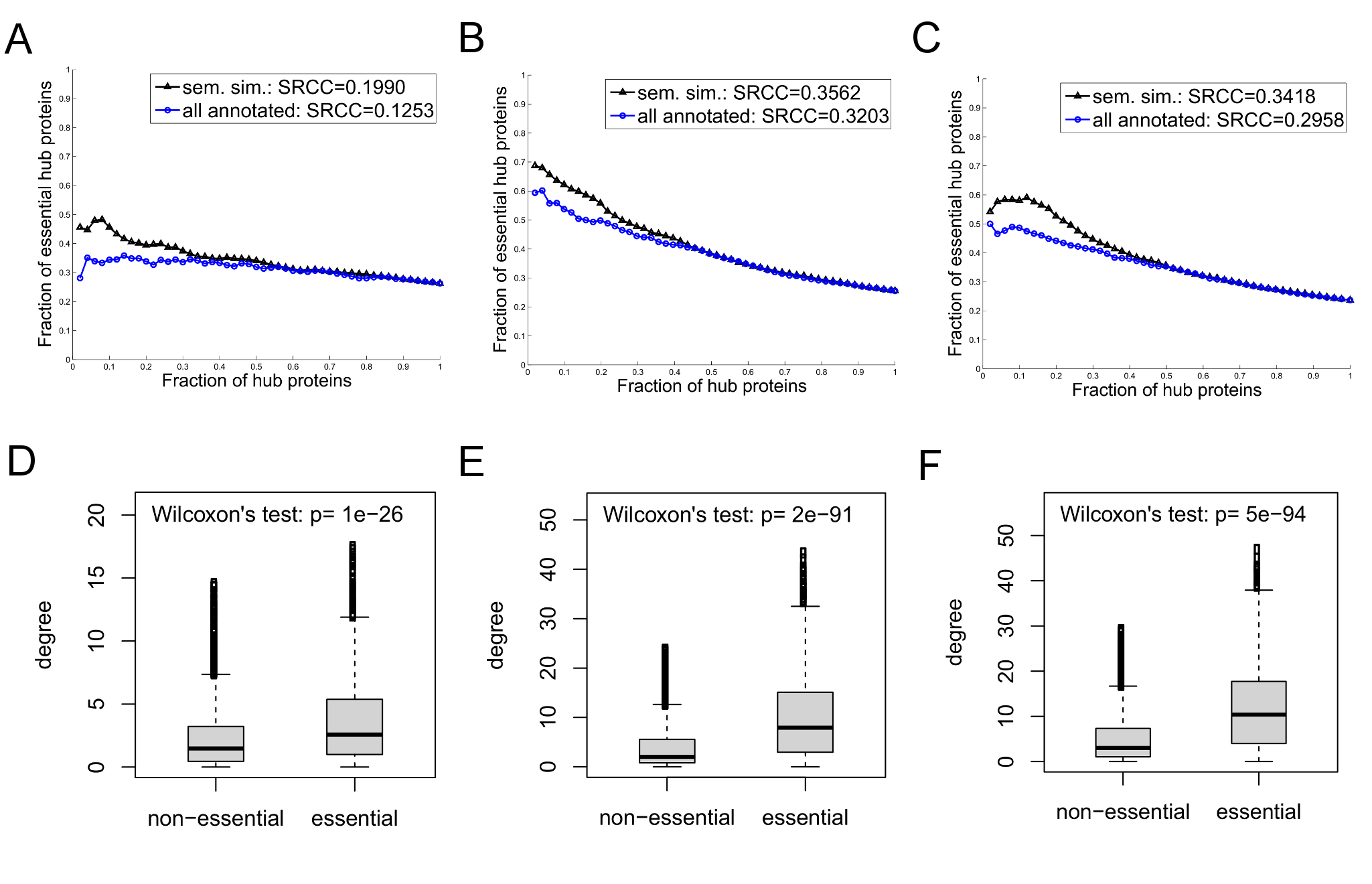

Supplement: Figure S3 — In all three networks, the semantic similarity degree is more correlated with protein essentiality than the overall interaction degree. (a)–(c) The fraction of essential proteins among hub proteins as more proteins are considered hub proteins for the Direct, Pull-down and Full networks; proteins are added in groups of 50 in a non-increasing order of the semantic similarity degree. For each network, the SRCC is computed between protein essentiality and either semantic similarity or all annotated degree; these values are boxed in each panel. (d)–(f) The semantic similarity weighted degree distribution of non-essential proteins is compared to that of essential proteins for the Direct, Pull-down and Full networks. (TIFF) [file pcbi.1002910.s003.tiff]

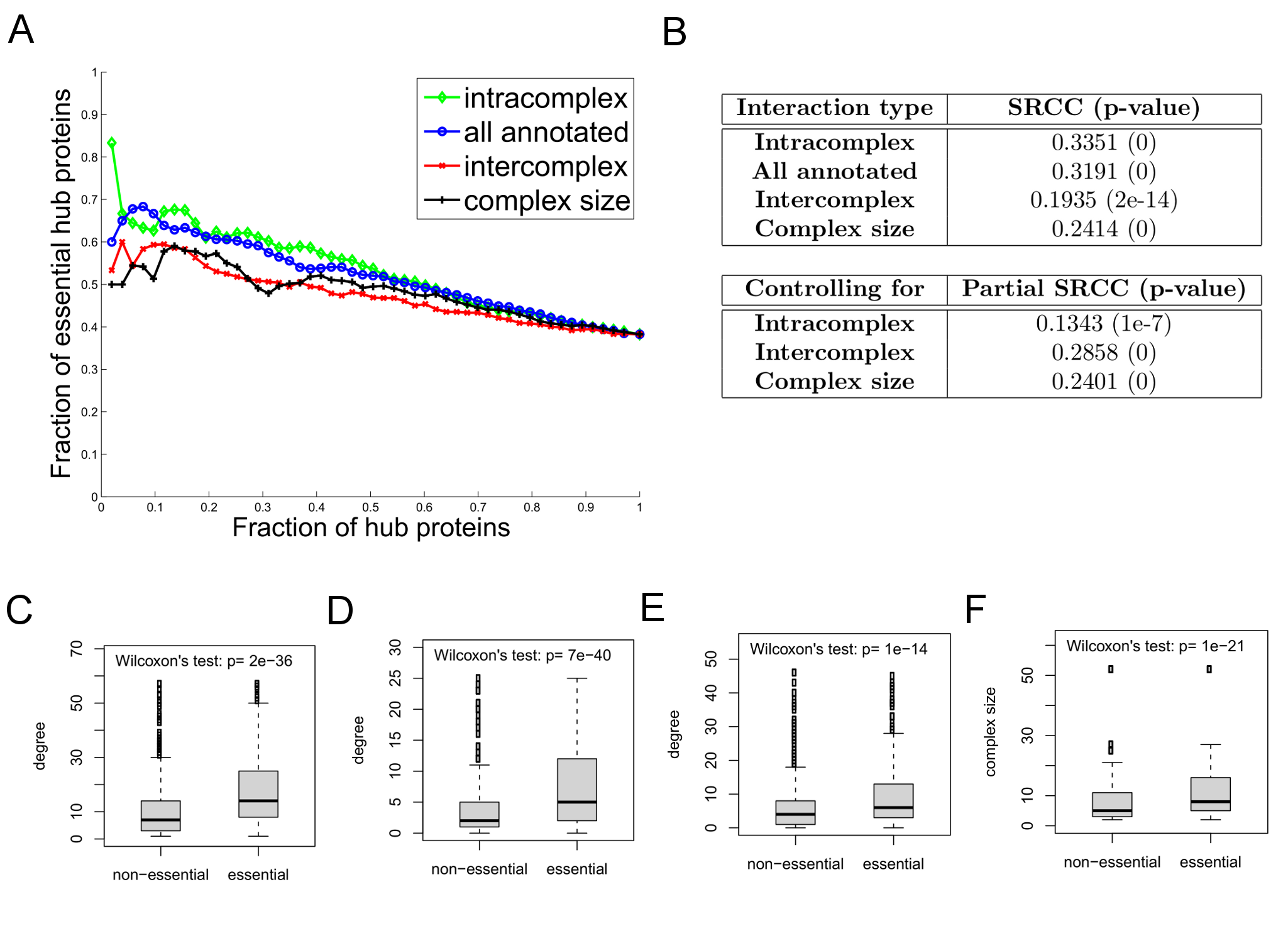

Supplement: Figure S4 — The intracomplex interaction degree is more correlated with protein essentiality than the overall interaction degree for proteins in the Pull-down network, when interactions are categorized using protein complexes. (a) The fraction of essential proteins among hub proteins as more proteins are considered hub proteins; proteins are added in groups of 50 in a non-increasing order of the interaction degree or the size of the largest complex to which the protein belongs. The relationship between protein essentiality and interaction degree is shown for intracomplex interactions (green), all annotated interactions (blue) and intercomplex interactions (red). The relationship between protein essentiality and complex size is also shown (black). (b) The correlations measured by SRCCs between essentiality and either intracomplex degree, all annotated degree, intercomplex degree, or complex size. The SRCC is highest between essentiality and intracomplex degree. The partial correlation is also computed between all annotated degree and essentiality when controlling for either intracomplex degree, intercomplex degree, or the size of the largest complex to which the protein belongs. Starred -values indicate those with values . (c)–(f) The degree distribution of non-essential proteins is compared to that of essential proteins within complexes for (c) all annotated degree, (d) intracomplex degree, (e) intercomplex degree, and (f) complex size. In each box plot, the horizontal bar within a box corresponds to the median of the distribution; the two ends of the box indicate the first and third quartiles; and the small circles show outliers within the 2–98th percentile range. The significance of the difference between the two degree distributions is measured by the Wilcoxon rank sum test. (TIFF) [file pcbi.1002910.s004.tiff]

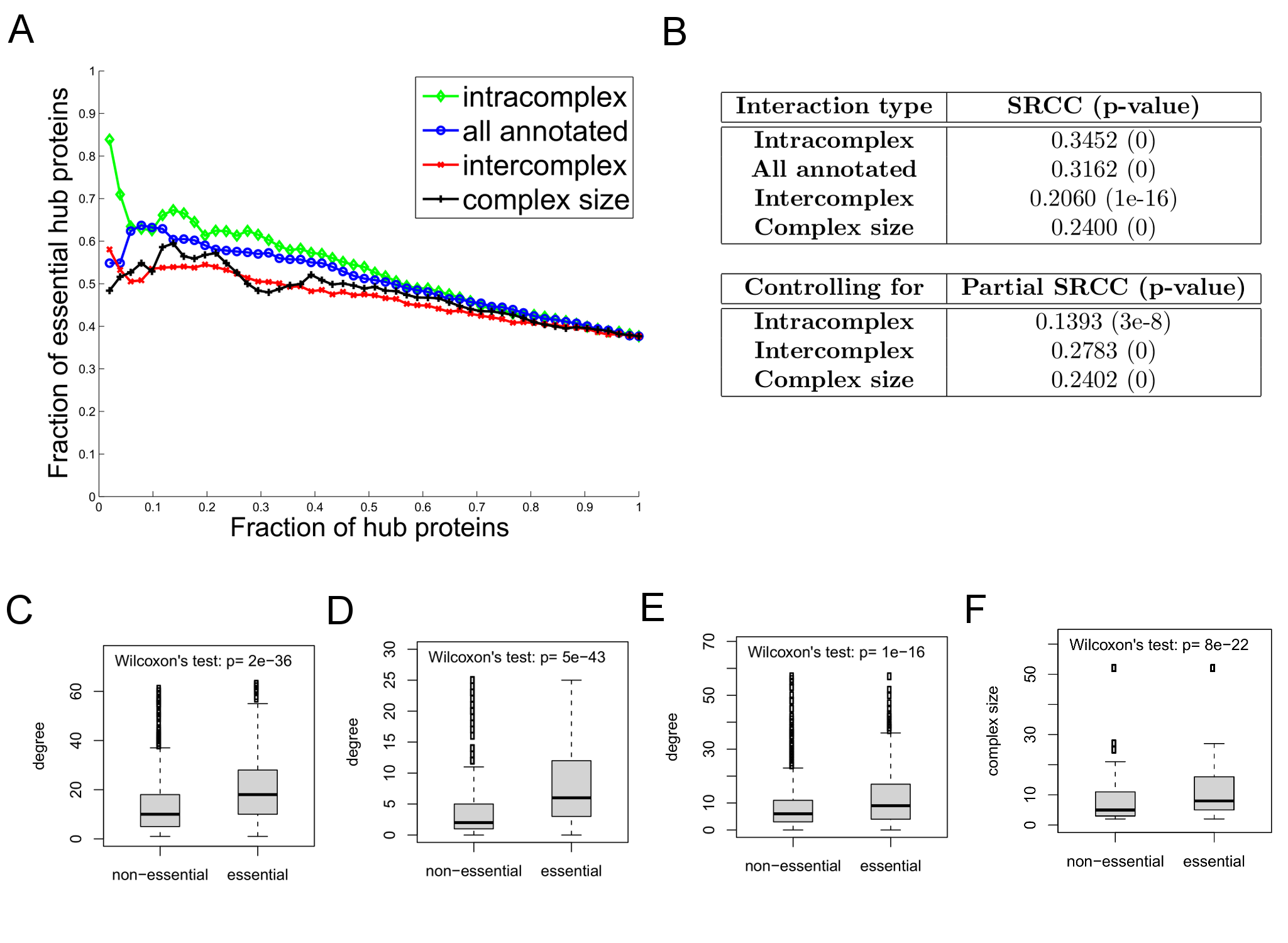

Supplement: Figure S5 — The intracomplex interaction degree is more correlated with protein essentiality than the overall interaction degree for proteins in the Full network, when interactions are categorized using protein complexes. (a) The fraction of essential proteins among hub proteins as more proteins are considered hub proteins; proteins are added in groups of 50 in a non-increasing order of the interaction degree or the size of the largest complex to which the protein belongs. The relationship between protein essentiality and interaction degree is shown for intracomplex interactions (green), all annotated interactions (blue) and intercomplex interactions (red). The relationship between protein essentiality and complex size is also shown (black). (b) The correlations measured by SRCCs between essentiality and either intracomplex degree, all annotated degree, intercomplex degree, or complex size. The SRCC is highest between essentiality and intracomplex degree. The partial correlation is also computed between all annotated degree and essentiality when controlling for either intracomplex degree, intercomplex degree, or the size of the largest complex to which the protein belongs. Starred -values indicate those with values . (c)–(f) The degree distribution of non-essential proteins is compared to that of essential proteins within complexes for (c) all annotated degree, (d) intracomplex degree, (e) intercomplex degree, and (f) complex size. In each box plot, the horizontal bar within a box corresponds to the median of the distribution; the two ends of the box indicate the first and third quartiles; and the small circles show outliers within the 2–98th percentile range. The significance of the difference between the two degree distributions is measured by the Wilcoxon rank sum test. (TIFF) [file pcbi.1002910.s005.tiff]

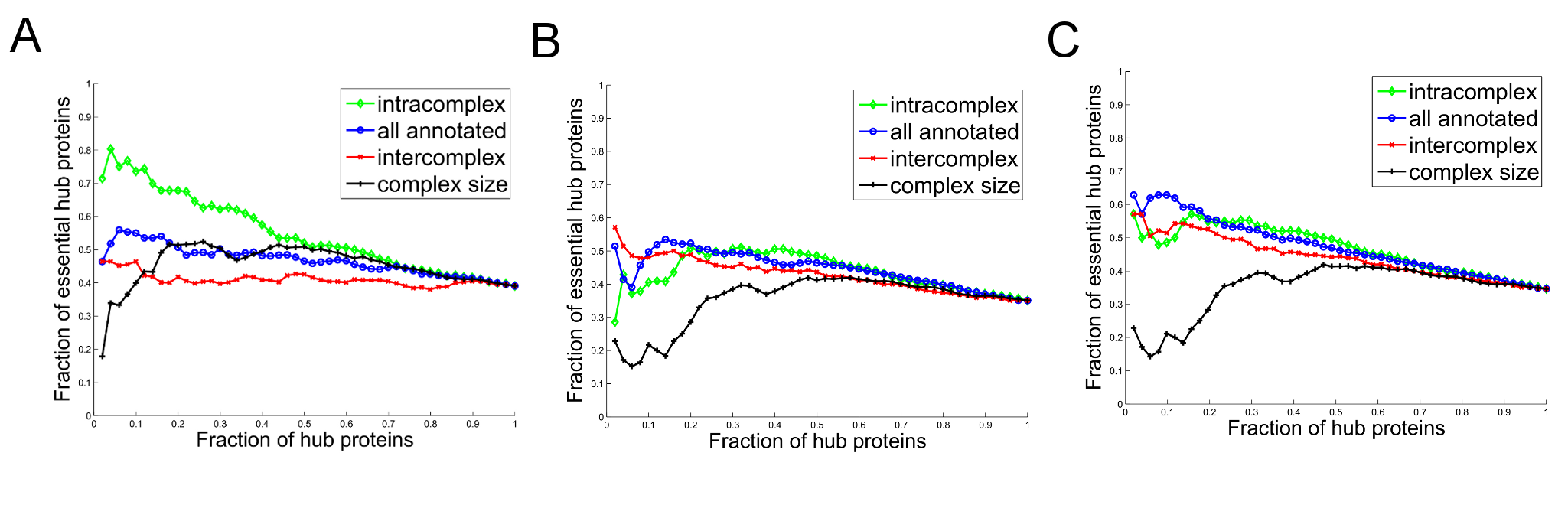

Supplement: Figure S6 — The correlations between interaction degree and essentiality for proteins in all complexes, including ribosomal complexes, for (a) Direct , (b) Pull-down and (c) Full networks. Interactions are categorized using protein complexes including ribosomal complexes. The fraction of essential proteins among hub proteins as more proteins are considered hub proteins; proteins are added in groups of 50 in a non-increasing order of the interaction degree or the size of the largest complex to which the protein belongs. The relationship between protein essentiality and interaction degree is shown for intracomplex interactions (green), all annotated interactions (blue) and intercomplex interactions (red). The relationship between protein essentiality and complex size is also shown (black). (TIFF) [file pcbi.1002910.s006.tiff]

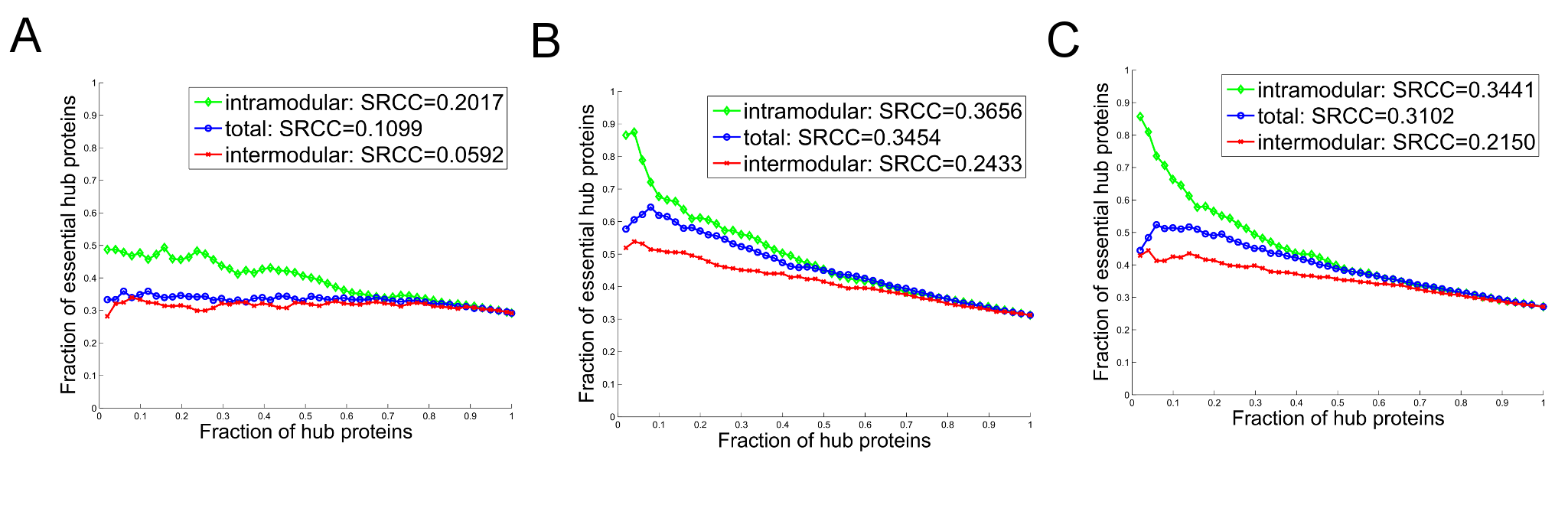

Supplement: Figure S7 — Essentiality is more correlated with intramodular interaction degree than it is with intermodular or total degree, when modules are determined in each network ((a) Direct , (b) Pull-down and (c) Full ) via network clustering approaches. To obtain clusters, we used the SPICi clustering algorithm, a local clustering approach, with a density threshold of 0.5 and a minimum increment ratio of 0.3. The fraction of essential proteins among hub proteins as more proteins are considered hub proteins; proteins are added in groups of 50 in a non-increasing order of the interaction degree. The relationship between protein essentiality and interaction degree is shown for intramodular interactions (green), total interactions (blue) and intermodular interactions (red). (TIFF) [file pcbi.1002910.s007.tiff]

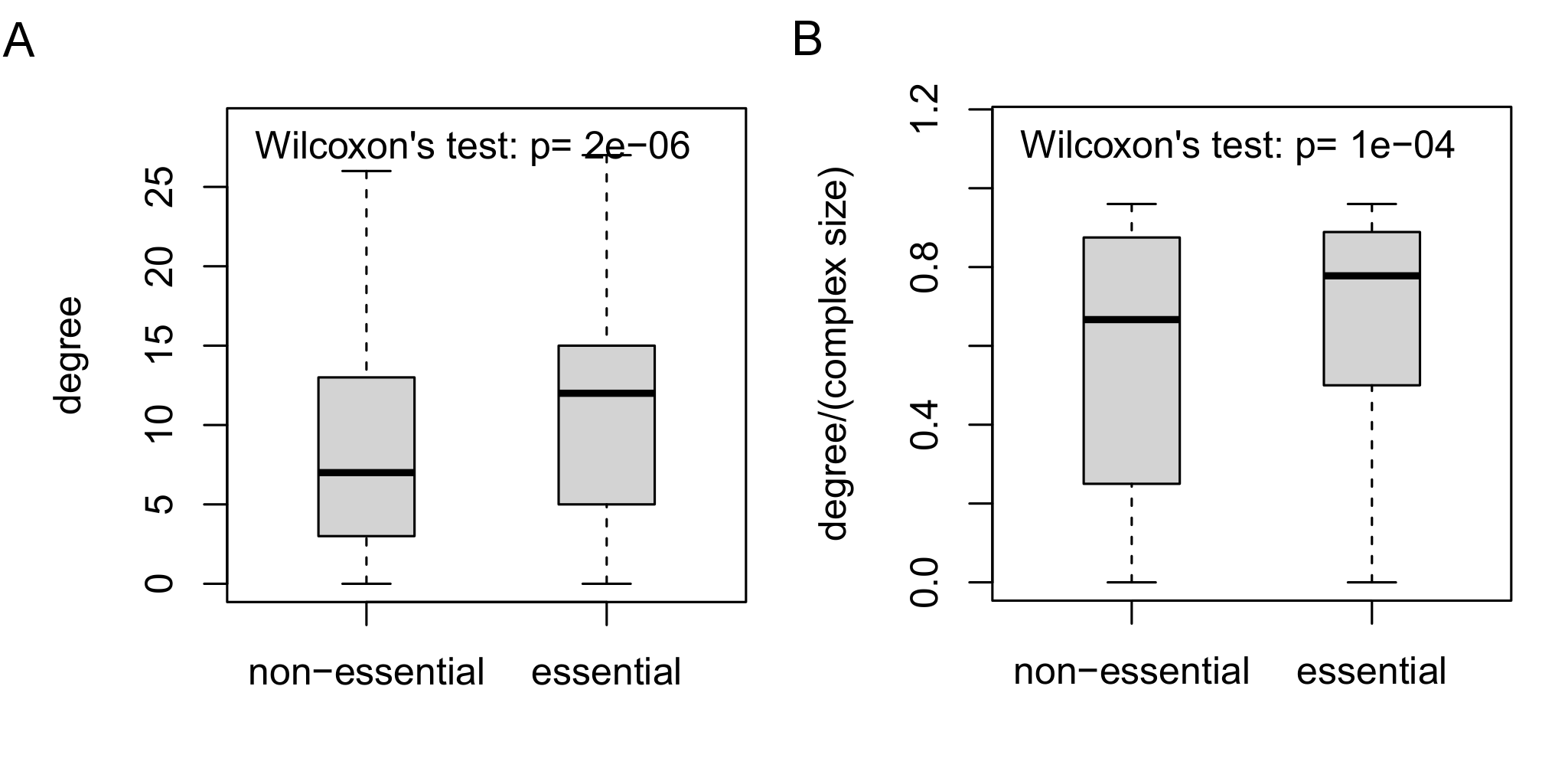

Supplement: Figure S8 — Within essential complexes, essential proteins tend to have a higher intracomplex degree in the Pull-down network than non-essential proteins. (a) The intracomplex degree or (b) the normalized intracomplex degree of essential proteins is significantly larger than that of non-essential proteins. Only protein complexes that have at least two essential proteins and at least two non-essential proteins, each with intracomplex interactions are considered. Outliers within the 2–98th percentiles are shown. The significance of the difference of the two degree distributions is determined by the Wilcoxon rank sum test. (TIFF) [file pcbi.1002910.s008.tiff]

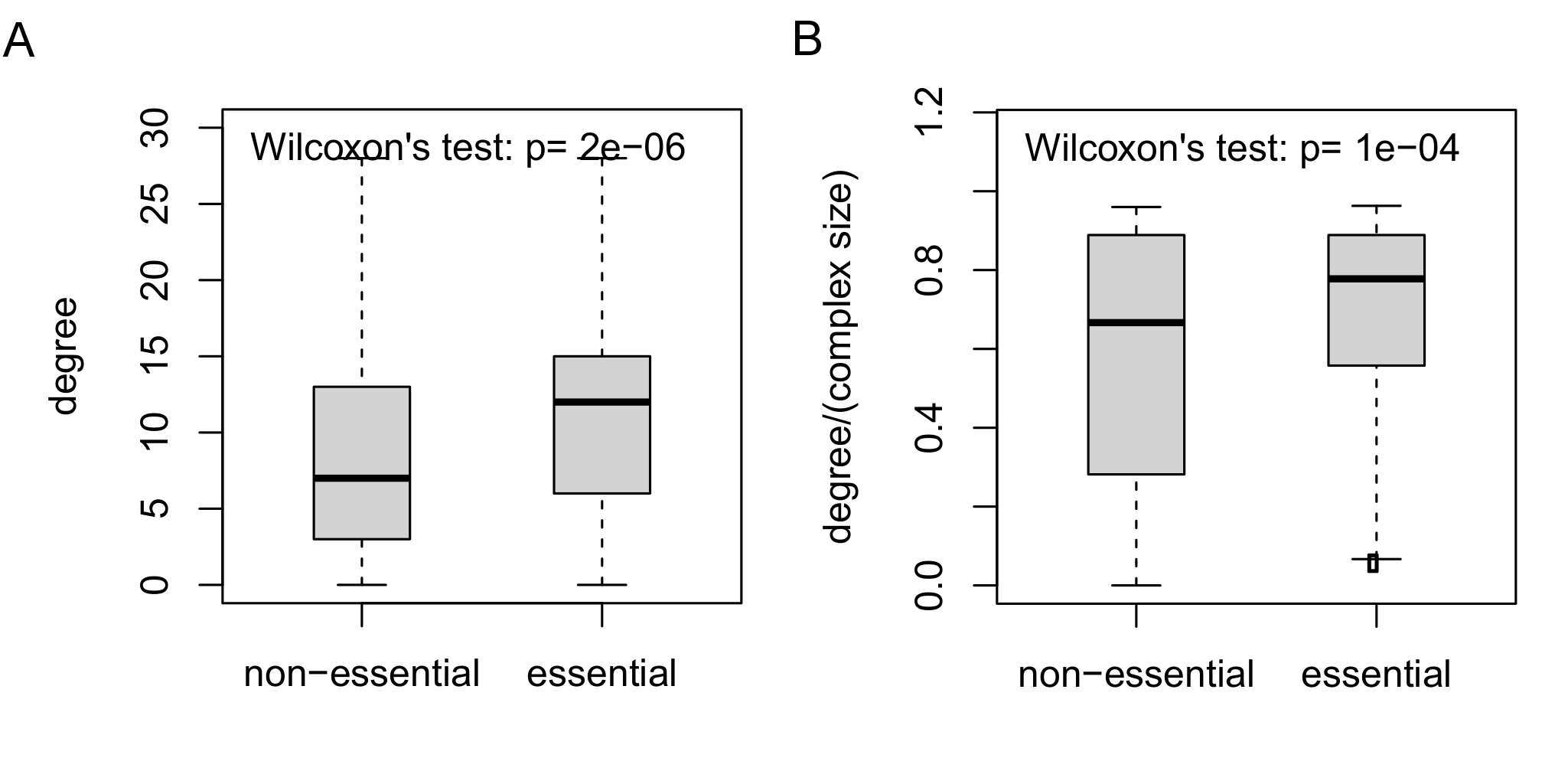

Supplement: Figure S9 — Within essential complexes, essential proteins tend to have a higher intracomplex degree in the Full network than non-essential proteins. (a) The intracomplex degree or (b) the normalized intracomplex degree of essential proteins is significantly larger than that of non-essential proteins. Only protein complexes that have at least two essential proteins and at least two non-essential proteins, each with intracomplex interactions are considered. Outliers within the 2–98th percentiles are shown. The significance of the difference of the two degree distributions is determined by the Wilcoxon rank sum test. (TIFF) [file pcbi.1002910.s009.tiff]

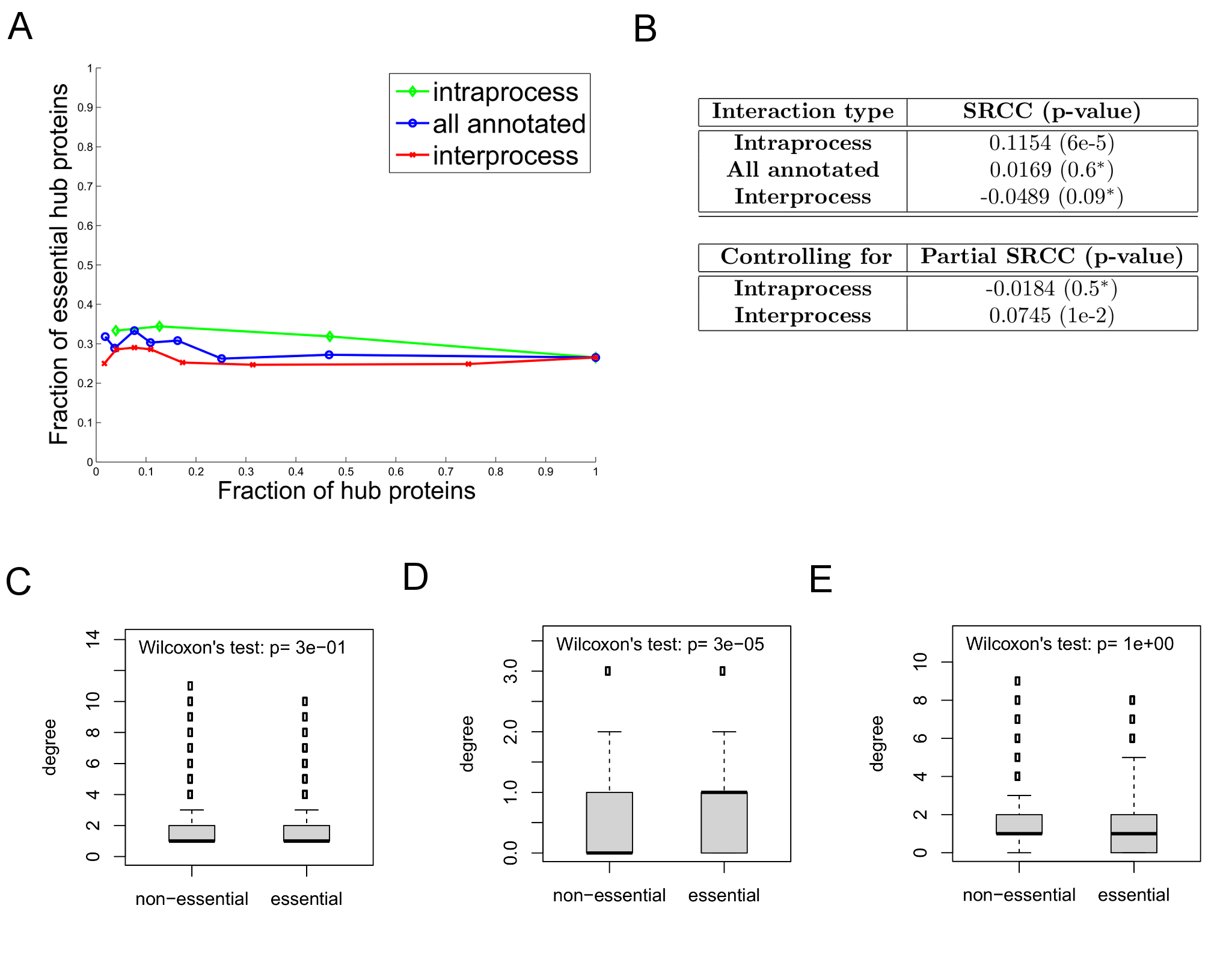

Supplement: Figure S10 — The intraprocess interaction degree is more correlated with protein essentiality than the overall interaction degree for proteins in the Y2H-union network, when interactions are categorized with specific GO BP terms, each of which annotates at most 50 proteins. (a) The fraction of essential proteins among hub proteins as more proteins are considered hub proteins; proteins are added in groups of size 20 (or larger so as to put proteins with the same degree in the same group). This fraction is highest for intraprocess degree (green), followed by all annotated degree (blue) and then by interprocess degree (red). (b) The correlations measured by SRCCs between essentiality and either intraprocess, all annotated or interprocess degree. The SRCC is highest between essentiality and intraprocess degree. The partial correlation is also computed between all annotated degree and essentiality when controlling for either intraprocess or interprocess degree. Starred -values indicate those with values . (c)–(e) The degree distribution of non-essential proteins is compared to that of essential proteins for (c) all annotated, (d) intraprocess, and (e) interprocess degree, respectively. In each box plot, the horizontal bar within a box corresponds to the median of the distribution; the two ends of the box indicate the first and third quartiles; and the small circles show outliers within the 2–98th percentile range. The significance of the difference between the two degree distributions is measured by the Wilcoxon rank sum test. For the Y2H-union network, essentiality and intraprocess degree have a small but statistically significant correlation. Essentiality is not significantly correlated with overall degree and interprocess degree. Further, essential proteins have higher average intraprocess degree than non-essential proteins in this network (panel (d)), while there is not a significant difference in all annotated degree or interprocess degree (panels (c) and (e)). (TIFF) [file pcbi.1002910.s010.tiff]

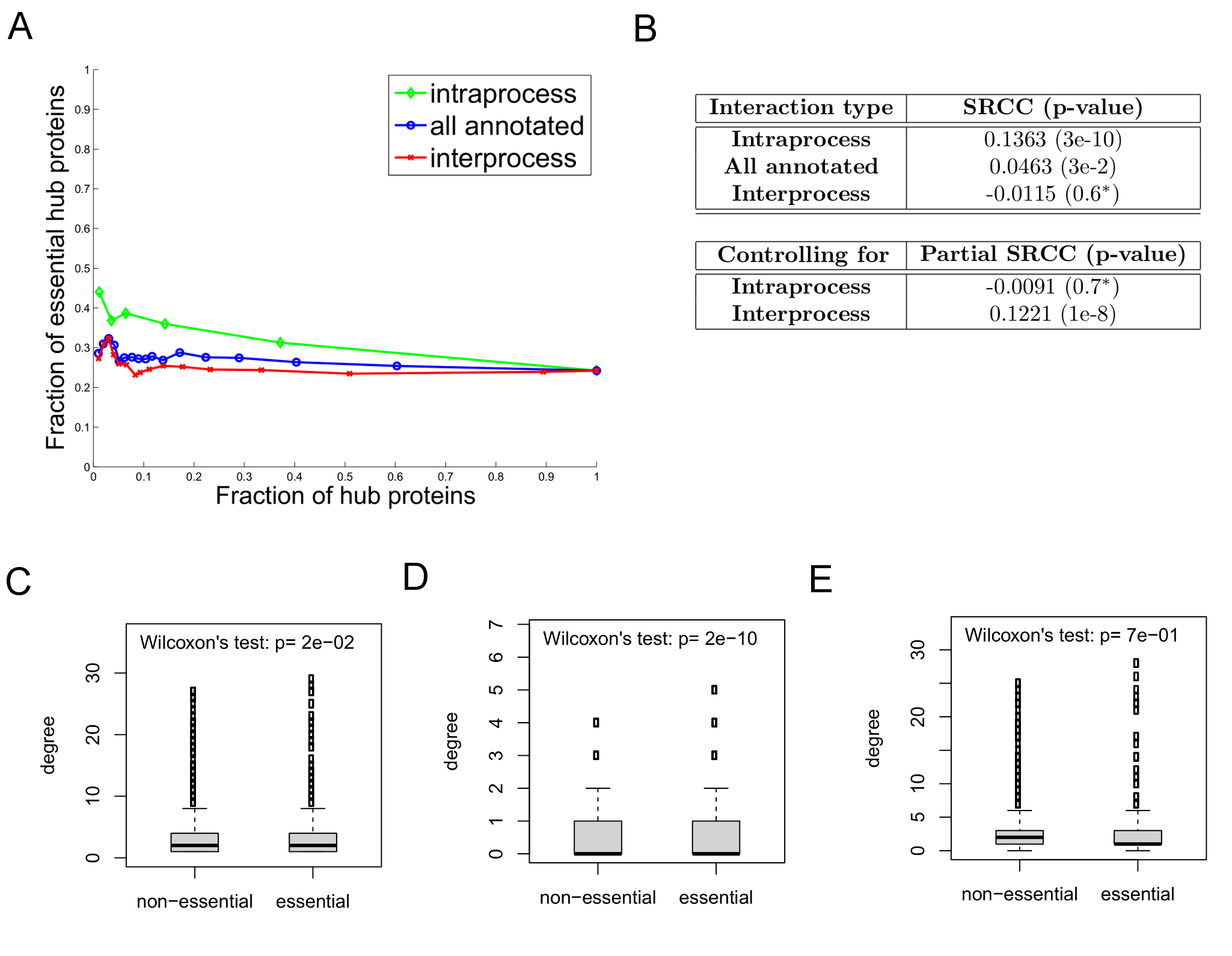

Supplement: Figure S11 — The intraprocess interaction degree is more correlated with protein essentiality than the overall interaction degree for proteins in the BinaryHQHT network, when interactions are categorized with specific GO BP terms, each of which annotates at most 50 proteins. (a) The fraction of essential proteins among hub proteins as more proteins are considered hub proteins; proteins are added in groups of size 20 (or larger so as to put proteins with the same degree in the same group). This fraction is highest for intraprocess degree (green), followed by all annotated degree (blue) and then by interprocess degree (red). (b) The correlations measured by SRCCs between essentiality and either intraprocess, all annotated or interprocess degree. The SRCC is highest between essentiality and intraprocess degree. The partial correlation is also computed between all annotated degree and essentiality when controlling for either intraprocess or interprocess degree. Starred -values indicate those with values . (c)–(e) The degree distribution of non-essential proteins is compared to that of essential proteins for (c) all annotated, (d) intraprocess, and (e) interprocess degree, respectively. In each box plot, the horizontal bar within a box corresponds to the median of the distribution; the two ends of the box indicate the first and third quartiles; and the small circles show outliers within the 2–98th percentile range. The significance of the difference between the two degree distributions is measured by the Wilcoxon rank sum test. For the BinaryHQHT network, essentiality and intraprocess degree have a small but statistically significant correlation. Essentiality has a smaller correlation with overall degree and is not correlated with interprocess degree. Further, essential proteins have higher average intraprocess degree than non-essential proteins in this network (panel (d)), while there is not a significant difference in interprocess degree (panel (e)). (TIFF) [file pcbi.1002910.s011.tiff]

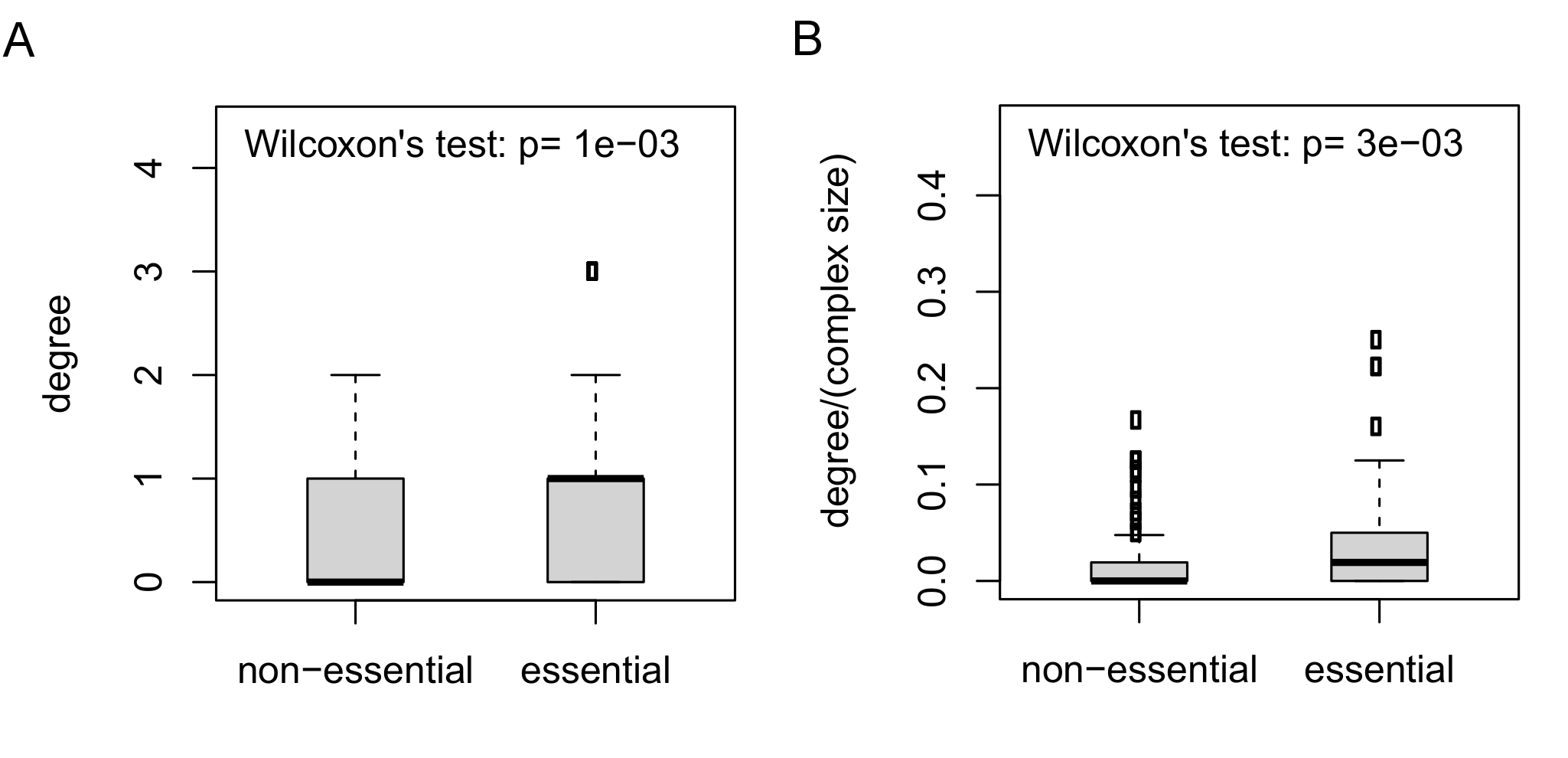

Supplement: Figure S12 — Essential proteins tend to have a higher intracomplex degree than non-essential proteins within protein complexes in the Y2H-union network. (a) The intracomplex degree or (b) the normalized intracomplex degree of essential proteins is significantly greater than that of non-essential proteins. Only protein complexes that have at least two essential proteins and at least two non-essential proteins with intracomplex interactions are tested. Outliers within 2–98% are shown. The significance of the difference of the two degree distributions is determined by the Wilcoxon rank sum test. (TIFF) [file pcbi.1002910.s012.tiff]

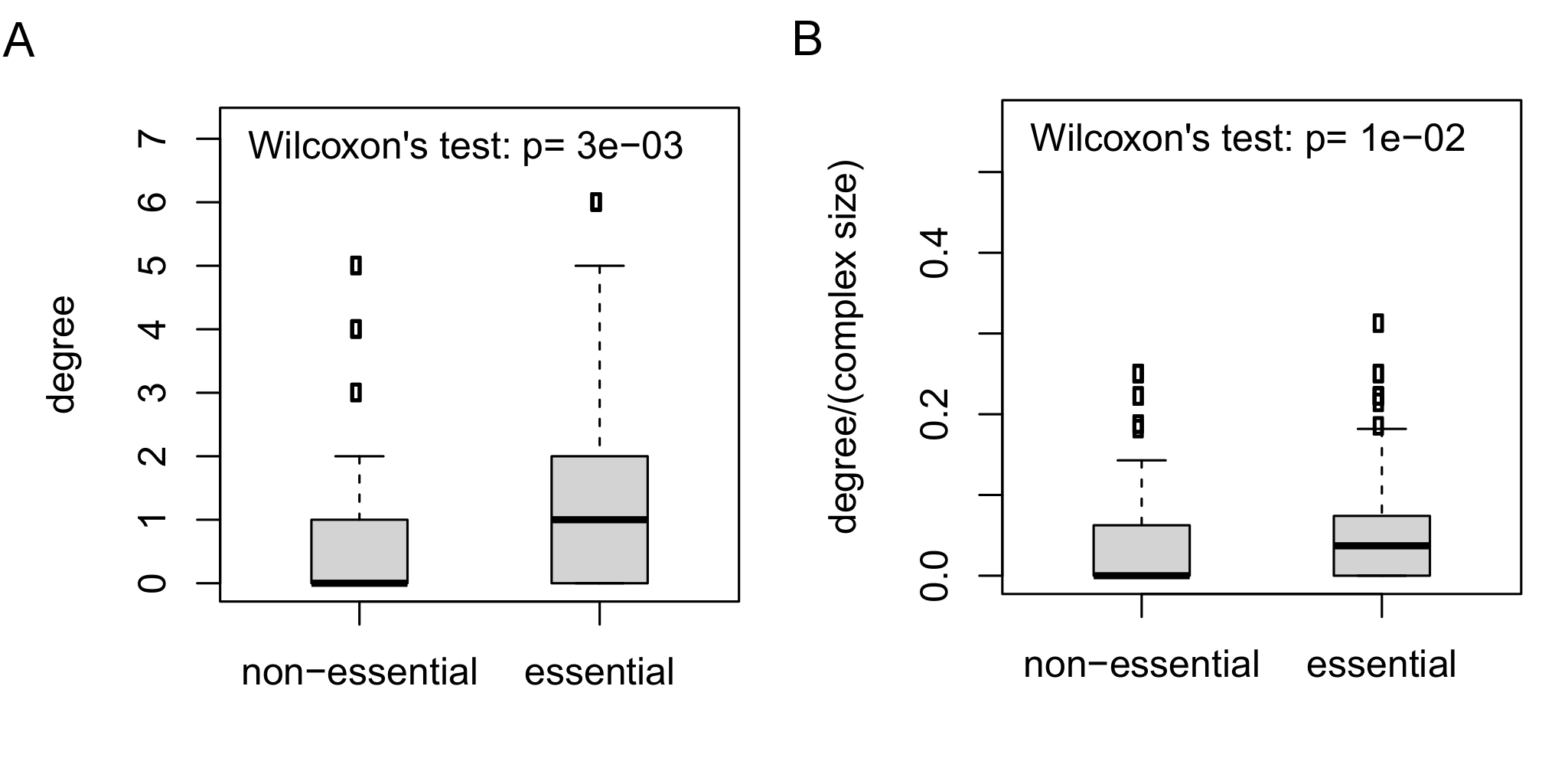

Supplement: Figure S13 — Essential proteins tend to have a higher intracomplex degree than non-essential proteins within protein complexes in the BinaryHQHT network. (a) The intracomplex degree or (b) the normalized intracomplex degree of essential proteins is significantly greater than that of non-essential proteins. Only protein complexes that have at least two essential proteins and at least two non-essential proteins with intracomplex interactions are tested. Outliers within 2–98% are shown. The significance of the difference of the two degree distributions is determined by the Wilcoxon rank sum test. (TIFF) [file pcbi.1002910.s013.tiff]

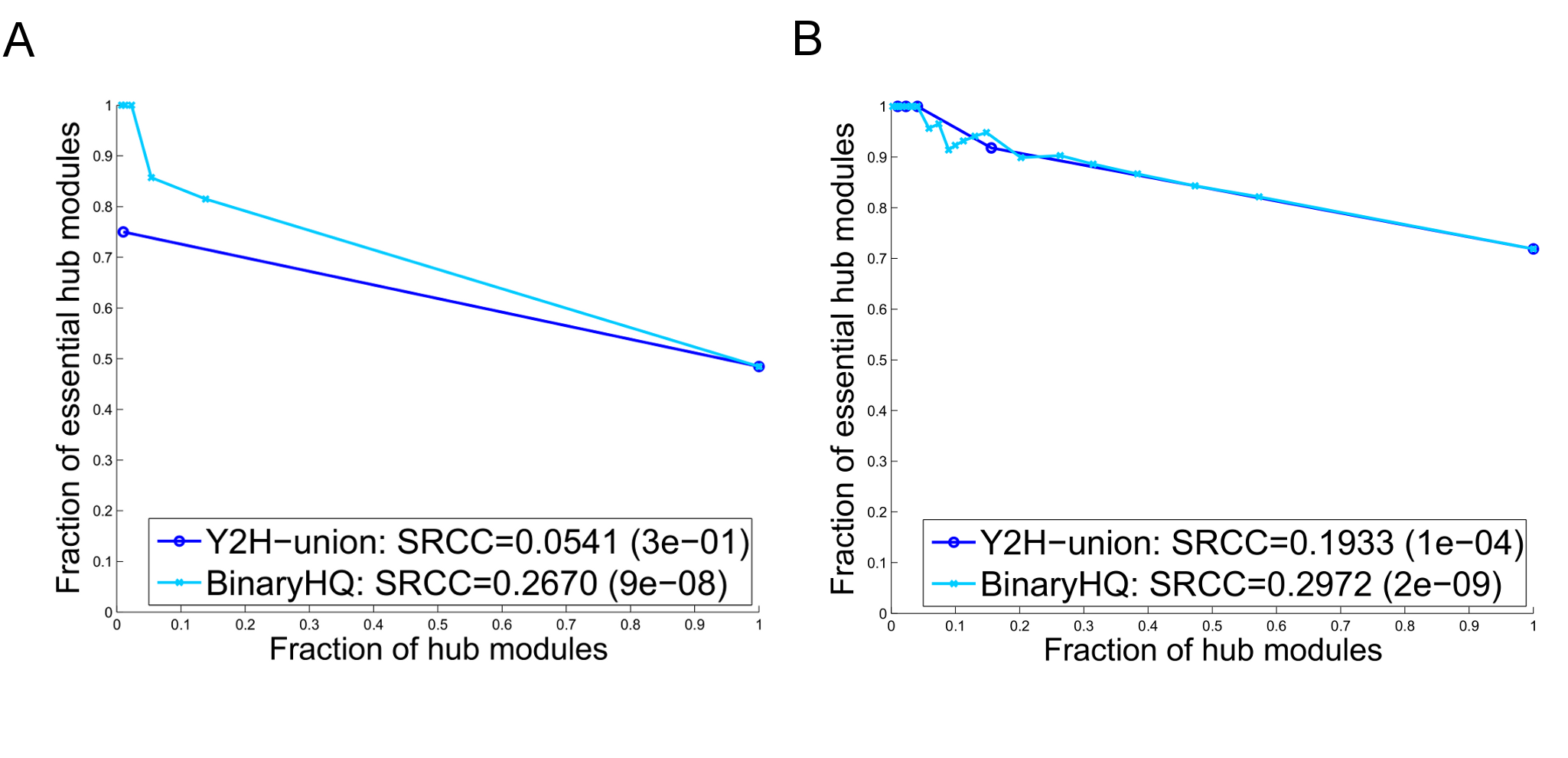

Supplement: Figure S14 — Cross-talk degree in a module-level network is correlated with module essentiality. For either (a) protein complexes or (b) filtered biological processes, the fraction of modules containing at least one essential protein among “hub modules” tends to decrease in each network as more modules are considered hubs. For the data shown, modules are added in a non-increasing order of cross-talk degree in the Y2H-union (blue) and BinaryHQHT (cyan) networks. Correlations between the binary essentiality of a module and its inferred cross-talk degree are computed using the SRCC and are shown for each network. The binary essentiality for a module is 1 if the module has at least one essential protein, and 0 otherwise. For the Y2H-union module network comprised of complexes (panel (a)), the correlation is not significant as we uncover only four complexes with crosstalks in this small network (549 edges, see Table S10). (TIFF) [file pcbi.1002910.s014.tiff]

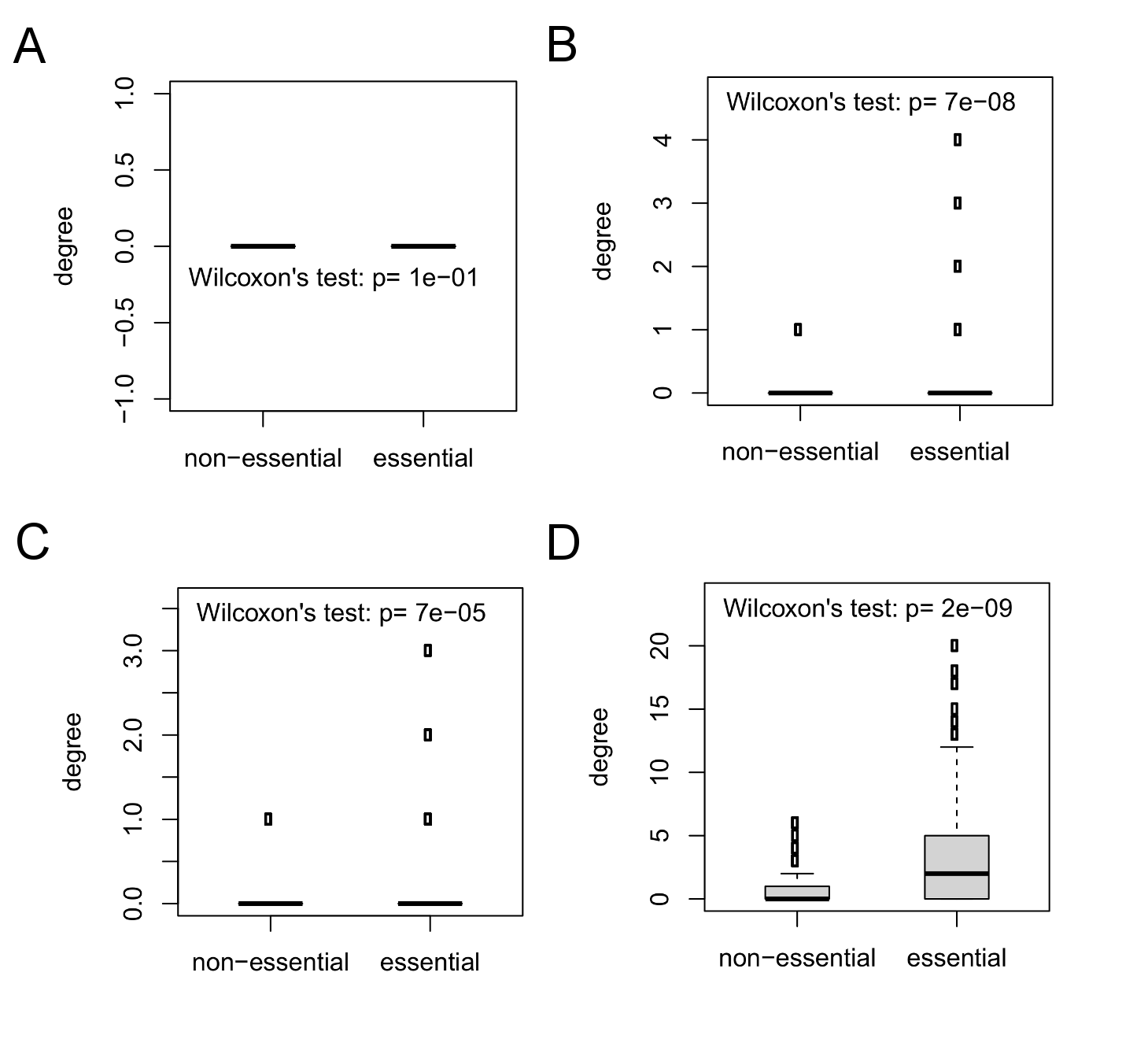

Supplement: Figure S15 — The cross-talk degree distribution of non-essential modules is compared to that of essential modules in the (a) Y2H-union and (b) BinaryHQHT networks for protein complexes and (c) Y2H-union and (d) BinaryHQHT networks for filtered biological processes. For the Y2H-union and BinaryHQHT networks, modules (derived from biological processes) with essential proteins have significantly higher cross-talk degree than modules without essential proteins, as determined by the Wilcoxon rank sum test. For modules derived from complexes in the Y2H-union network, the differences between essential and non-essential modules are not significant, as there are only four modules for which we can uncover cross-talks. (TIFF) [file pcbi.1002910.s015.tiff]

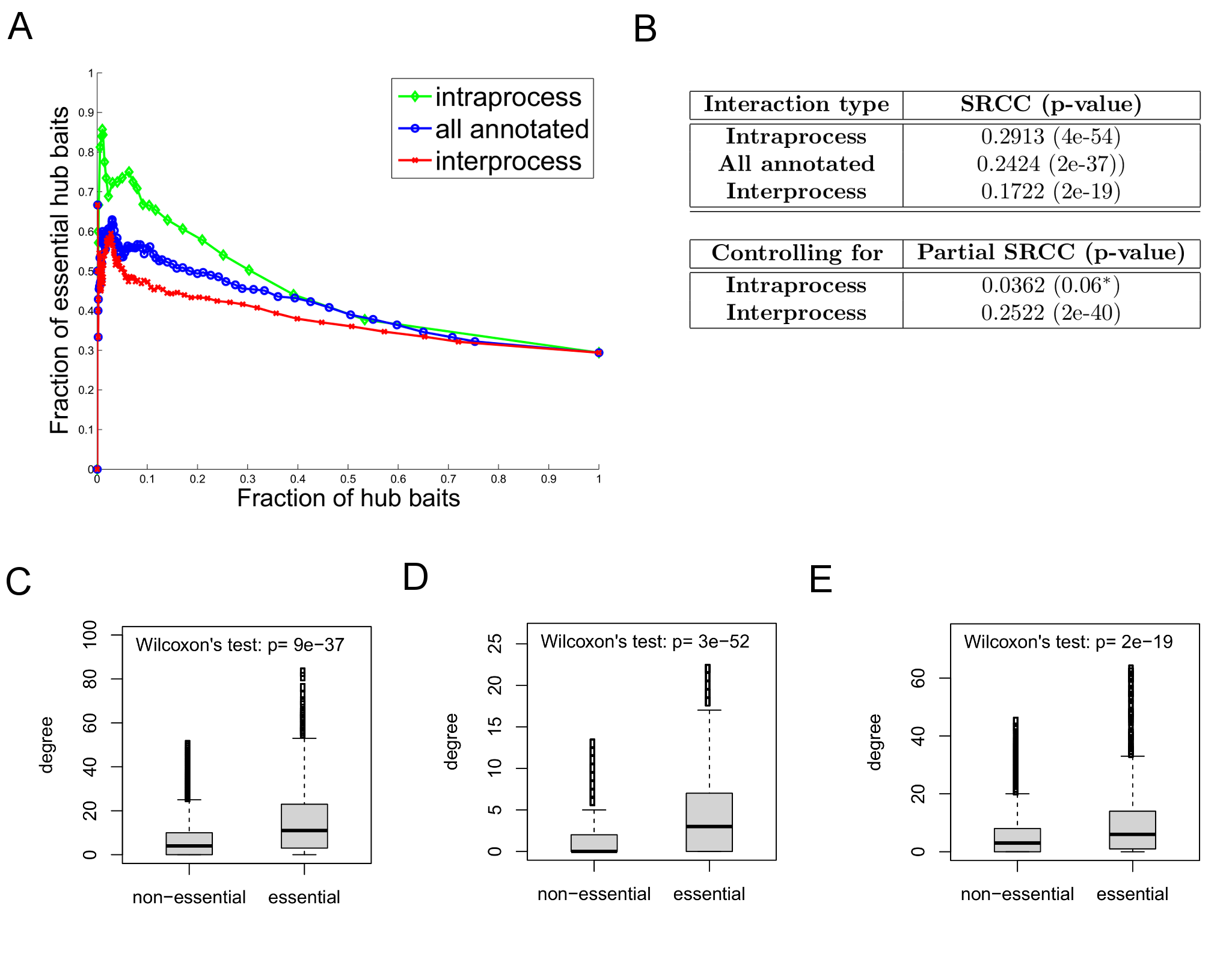

Supplement: Figure S16 — The intraprocess interaction degree is more correlated with protein essentiality than the overall interaction degree for bait proteins in the Pull-downf network excluding small-scale experiments, when interactions are categorized with specific GO BP terms, each of which annotates at most 50 proteins. All tests were done for only bait proteins in the Pull-downf network. (a) The fraction of essential proteins among hub proteins as more proteins are considered hub proteins; proteins are added in a non-increasing order of the interaction degree. This fraction is highest for intraprocess degree (green), followed by all annotated degree (blue) and then by interprocess degree (red). (b) The correlations measured by SRCCs between essentiality and either intraprocess, all annotated or interprocess degree. The SRCC is highest between essentiality and intraprocess degree. The partial correlation is also computed between all annotated degree and essentiality when controlling for either intraprocess or interprocess degree. Starred -values indicate those with values . (c)–(e) The degree distribution of non-essential proteins is compared to that of essential proteins for (c) all annotated, (d) intraprocess, and (e) interprocess degree, respectively. In each box plot, the horizontal bar within a box corresponds to the median of the distribution; the two ends of the box indicate the first and third quartiles; and the small circles show outliers within the 2–98th percentile range. The significance of the difference between the two degree distributions is measured by the Wilcoxon rank sum test. (TIFF) [file pcbi.1002910.s016.tiff]

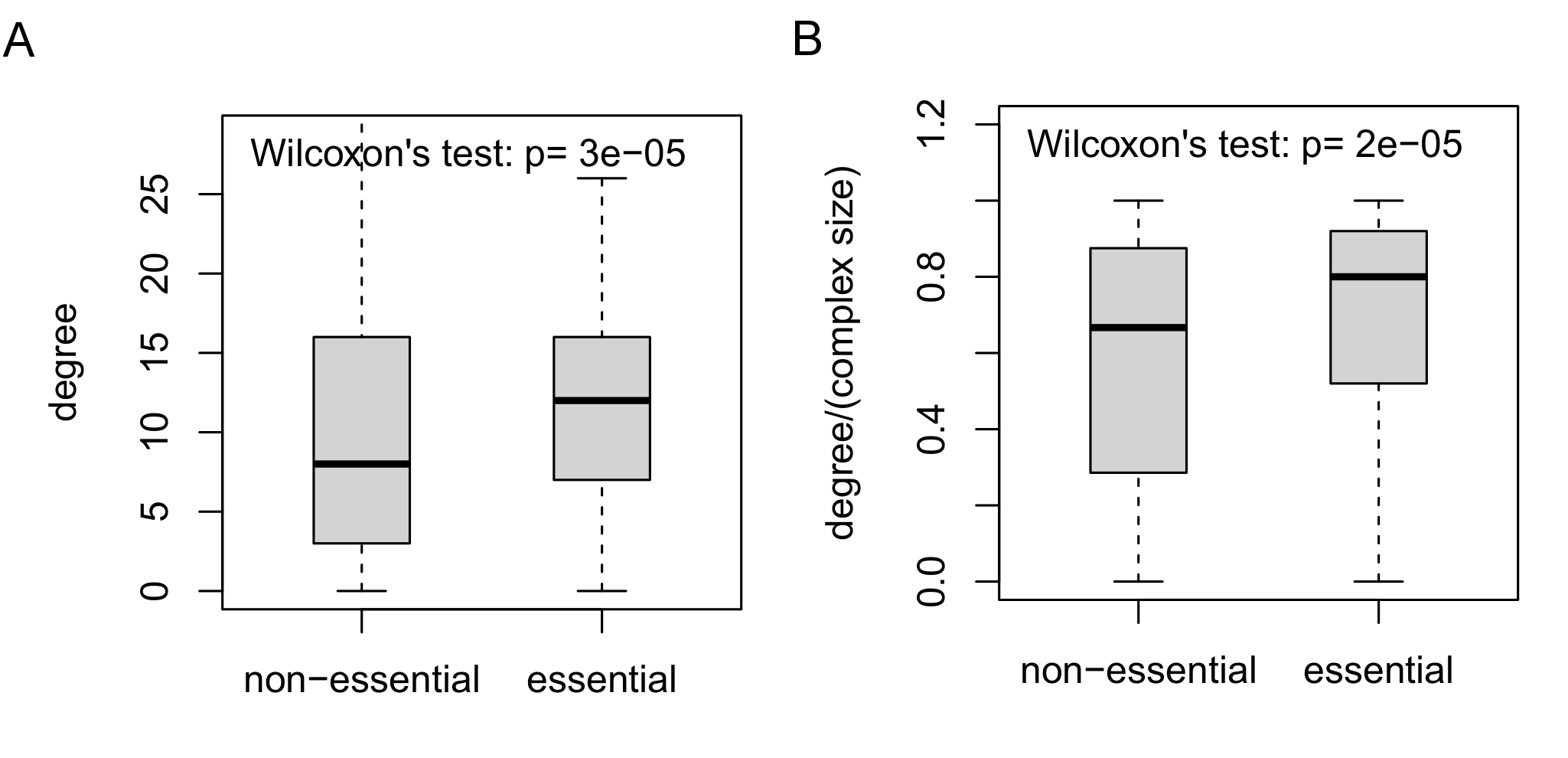

Supplement: Figure S17 — Within essential complexes, essential bait proteins tend to have a higher intracomplex degree in the Pull-downf network than non-essential bait proteins. (a) The intracomplex degree or (b) the normalized intracomplex degree of essential bait proteins is significantly larger than that of non-essential bait proteins. Only protein complexes that have at least two essential bait proteins and at least two non-essential bait proteins, each with intracomplex interactions, are considered. Outliers within the 2–98th percentiles are shown. The significance of the difference between the two degree distributions is determined by the Wilcoxon rank sum test. (TIFF) [file pcbi.1002910.s017.tiff]
