# Supplementary material for: From Hub Proteins to Hub Modules: The Relationship Between Essentiality and Centrality in the Yeast Interactome at Different Scales of Organization
Source: PLoS Comput Biol. 2013 Feb 21;9(2):e1002910. doi: 10.1371/journal.pcbi.1002910 (PMC3578755; doi:10.1371/journal.pcbi.1002910)
Supplement: Table S1 — The number of proteins, the number of interactions and the fraction of essential proteins for each of the three physical interaction networks considered. (PDF) [file pcbi.1002910.s018.pdf]

Network	Num Proteins	Num Interactions	Fraction of Essential Proteins
**Direct**	4031	15073	0.22
**Pull-down**	4449	36455	0.22
**Full**	5167	50170	0.20

Table S 1. The number of proteins, the number of interactions and the fraction of essential proteins for each of the three physical interaction networks considered.
