# Supplementary material for: From Hub Proteins to Hub Modules: The Relationship Between Essentiality and Centrality in the Yeast Interactome at Different Scales of Organization
Source: PLoS Comput Biol. 2013 Feb 21;9(2):e1002910. doi: 10.1371/journal.pcbi.1002910 (PMC3578755; doi:10.1371/journal.pcbi.1002910)
Supplement: Table S2 — A substantial fraction of physical interactions in the Direct network are intermodular. (PDF) [file pcbi.1002910.s019.pdf]

Modules	Num Annotated Interactions	Num Intramodular Interactions	Num Intermodular Interactions	Intermodular Percentage
Protein complexes	3825	1364	2461	64.34%
BPs, 50	10119	2530	7589	75.00%
BPs, 100	11757	3706	8051	68.48%
BPs, 300	13350	5780	7570	56.70%
BPs, 500	13573	6689	6884	50.72%
Filtered BPs	4415	575	3840	86.98%

Table S 2. A substantial fraction of physical interactions are intermodular. Modules gives the set of functional modules considered. These are: 1) protein complexes, 2) a subset of specific GO BP terms, each of which annotates at most 50, 100, 300, or 500 proteins in the yeast genome, or 3) a subset of filtered biological processes (as decribed in **Materials and Methods**). **Num Annotated Interactions** gives the number of interactions in the subnetwork generated from the *Direct* network where nodes represent proteins in the considered modules and edges represent interactions amongst them. **Num Intramodular Interactions** gives the number of interactions in the subnetwork where the two interacting proteins belong to the same module. **Num Intermodular Interactions** gives the number of interactions in the subnetwork where the two interacting proteins belong to different modules. **Intermodular Percentage** gives the percentage of intermodular interactions amongst the annotated interactions.
