# Supplementary material for: From Hub Proteins to Hub Modules: The Relationship Between Essentiality and Centrality in the Yeast Interactome at Different Scales of Organization
Source: PLoS Comput Biol. 2013 Feb 21;9(2):e1002910. doi: 10.1371/journal.pcbi.1002910 (PMC3578755; doi:10.1371/journal.pcbi.1002910)
Supplement: Table S3 — Correlation between cross-talk (CT) degree and binary module essentiality. (PDF) [file pcbi.1002910.s020.pdf]

(a) Protein complexes

**Network**	$\mathbf{SRCC_{degree}}$	$\mathbf{SRCC_{degree/size}}$	$\mathbf{SRCC_{degree \cdot size}}$
**Direct**	0.3358 (1e-11)	0.2735 (4e-08)	0.2233 (9e-06)
**Pull-down**	0.3917 (1e-15)	0.2923 (4e-09)	0.2729 (5e-08)
**Full**	0.4081 (0e+00)	0.2824 (1e-08)	0.2924 (4e-09)

(b) Filtered biological processes

**Network**	$\mathbf{SRCC_{degree}}$	$\mathbf{SRCC_{degree/size}}$	$\mathbf{SRCC_{degree \cdot size}}$
**Direct**	0.3400 (5e-12)	0.2262 (6e-06)	0.2326 (3e-06)
**Pull-down**	0.3866 (2e-15)	0.2656 (1e-07)	0.2838 (1e-08)
**Full**	0.3606 (2e-13)	0.1253 (1e-02)	0.2533 (4e-07)

Table S 3. Correlation between cross-talk (CT) degree and binary module essentiality for (a) protein complexes and (b) filtered biological processes. $\mathbf{SRCC_{degree}}$ gives the SRCC between cross-talk degree and essentiality. $\mathbf{SRCC_{degree/size}}$ gives the SRCC between the normalized cross-talk degree (i.e., cross-talk degree of the module divided by the module size) and essentiality. $\mathbf{SRCC_{degree \cdot size}}$ gives the partial SRCC between cross-talk degree and essentiality when controlling for the module size. *P*-values for the SRCCs are shown within parentheses. A module is considered essential if has at least one essential protein, and not essential otherwise.
