# Supplementary material for: From Hub Proteins to Hub Modules: The Relationship Between Essentiality and Centrality in the Yeast Interactome at Different Scales of Organization
Source: PLoS Comput Biol. 2013 Feb 21;9(2):e1002910. doi: 10.1371/journal.pcbi.1002910 (PMC3578755; doi:10.1371/journal.pcbi.1002910)
Supplement: Table S4 — Correlation between cross-talk (CT) degree and the fraction of essential proteins in the module. (PDF) [file pcbi.1002910.s021.pdf]

(a) Protein complexes

Network	$\mathbf{SRCC_{degree}}$	$\mathbf{SRCC_{degree/size}}$	$\mathbf{SRCC_{degree \cdot size}}$
**Direct**	0.2725 (5e-08)	0.2283 (5e-06)	0.1859 (2e-04)
**Pull-down**	0.3335 (1e-11)	0.2612 (2e-07)	0.2471 (8e-07)
**Full**	0.3321 (2e-11)	0.2369 (2e-06)	0.2446 (1e-06)

(b) Filtered biological processes

Network	$\mathbf{SRCC_{degree}}$	$\mathbf{SRCC_{degree/size}}$	$\mathbf{SRCC_{degree \cdot size}}$
**Direct**	0.1777 (4e-04)	0.1780 (4e-04)	0.1897 (2e-04)
**Pull-down**	0.2366 (2e-06)	0.2500 (6e-07)	0.2596 (2e-07)
**Full**	0.1751 (5e-04)	0.1486 (3e-03)	0.1884 (2e-04)

Table S 4. Correlation between cross-talk (CT) degree and the fraction of essential proteins in the module for (a) protein complexes and (b) filtered biological processes. Module essentiality is defined as the fraction of essential proteins within a module. $\mathbf{SRCC_{degree}}$ gives the SRCC between the cross-talk degree of a module and its fraction of essential proteins. $\mathbf{SRCC_{degree/size}}$ gives the SRCC between the normalized cross-talk degree (i.e., the cross-talk degree of the module divided by the module size) and essentiality fraction. $\mathbf{SRCC_{degree \cdot size}}$ gives the partial SRCC between cross-talk degree and essentiality fraction when controlling for the module size. P-values for the SRCCs are shown within parentheses.
