# Supplementary material for: From Hub Proteins to Hub Modules: The Relationship Between Essentiality and Centrality in the Yeast Interactome at Different Scales of Organization
Source: PLoS Comput Biol. 2013 Feb 21;9(2):e1002910. doi: 10.1371/journal.pcbi.1002910 (PMC3578755; doi:10.1371/journal.pcbi.1002910)
Supplement: Table S5 — Correlation between cross-talk (CT) degree and binary module essentiality after removing functionally similar cross-talks. (PDF) [file pcbi.1002910.s022.pdf]

(a) Protein complexes

Network	$\mathbf{SRCC_{degree}}$	$\mathbf{SRCC_{degree/size}}$	$\mathbf{SRCC_{degree \cdot size}}$
**Direct**	0.3112 (3e-10)	0.2795 (2e-08)	0.1974 (9e-05)
**Pull-down**	0.2933 (4e-09)	0.2294 (5e-06)	0.1753 (5e-04)
**Full**	0.3093 (4e-10)	0.2288 (5e-06)	0.1924 (1e-04)

(b) Filtered biological processes

Network	$\mathbf{SRCC_{degree}}$	$\mathbf{SRCC_{degree/size}}$	$\mathbf{SRCC_{degree \cdot size}}$
**Direct**	0.3325 (2e-11)	0.2186 (1e-05)	0.2220 (1e-05)
**Pull-down**	0.3786 (9e-15)	0.2608 (2e-07)	0.2734 (4e-08)
**Full**	0.3475 (2e-12)	0.1068 (3e-02)	0.2384 (2e-06)

Table S 5. Correlation between cross-talk (CT) degree and binary module essentiality after removing functionally similar cross-talks for (a) protein complexes and (b) filtered biological processes. We removed cross-talks between modules that are annotated with a shared general biological process (see main body of the paper). In this restricted module-level network, $\mathbf{SRCC_{degree}}$ gives the SRCC between the cross-talk degree and essentiality. $\mathbf{SRCC_{degree/size}}$ gives the SRCC between the normalized cross-talk degree (i.e., cross-talk degree of the module divided by the module size) and essentiality. $\mathbf{SRCC_{degree \cdot size}}$ gives the partial SRCC between cross-talk degree and essentiality when controlling for the module size. P-values for the SRCCs are shown within parentheses. A module is considered essential if has at least one essential protein, and not essential otherwise.
