# Supplementary material for: From Hub Proteins to Hub Modules: The Relationship Between Essentiality and Centrality in the Yeast Interactome at Different Scales of Organization
Source: PLoS Comput Biol. 2013 Feb 21;9(2):e1002910. doi: 10.1371/journal.pcbi.1002910 (PMC3578755; doi:10.1371/journal.pcbi.1002910)
Supplement: Table S6 — The significant correlation between cross-talk degree and binary module essentiality persists for a range of odd-scores in the Direct network. (PDF) [file pcbi.1002910.s023.pdf]

(a) Protein complexes

Odd-score cutoff	# Cross-talks	SRCC (p-value)
**1.5**	228	0.3430 (3e-12)
**2**	194	0.3358 (1e-11)
**3**	112	0.2841 (1e-08)
**4**	60	0.1978 (8e-05)
**5**	25	0.1046 (4e-02)

(b) Filtered biological processes

Odd-score cutoff	# Cross-talks	SRCC (p-value)
**1.5**	1955	0.3452 (2e-12)
**2**	1149	0.3400 (5e-12)
**3**	199	0.2155 (2e-05)
**4**	56	0.0885 (0.08*)
**5**	12	0.0350 (0.5*)

Table S 6. The significant correlation between cross-talk degree and binary module essentiality persists for a range of odd-scores in the *Direct* network for (a) protein complexes and (b) filtered biological processes. Odd-score cutoff gives the minimum odd-score for a module pair to be considered a cross-talk. **# Cross-talks** gives the number of cross-talks at each cutoff. **SRCC (p-value)** gives the Spearman's rho rank correlation coefficient between cross-talk degree and binary module essentiality. For larger odd-score cutoffs, there are fewer cross-talks and this results in somewhat lower SRCC values.
