# Supplementary material for: From Hub Proteins to Hub Modules: The Relationship Between Essentiality and Centrality in the Yeast Interactome at Different Scales of Organization
Source: PLoS Comput Biol. 2013 Feb 21;9(2):e1002910. doi: 10.1371/journal.pcbi.1002910 (PMC3578755; doi:10.1371/journal.pcbi.1002910)
Supplement: Table S7 — The significant correlation between cross-talk degree and binary module essentiality persists for a range of odd-scores in the Pull-down network. (PDF) [file pcbi.1002910.s024.pdf]

(a) Protein complexes

Odd-score cutoff	# Cross-talks	SRCC (p-value)
**1.5**	641	0.3985 (2e-16)
**2**	535	0.3917 (1e-15)
**3**	311	0.3363 (9e-12)
**4**	189	0.2845 (1e-08)
**5**	125	0.2764 (3e-08)

(b) Filtered biological processes

Odd-score cutoff	# Cross-talks	SRCC (p-value)
**1.5**	2400	0.3910 (1e-15)
**2**	1409	0.3866 (2e-15)
**3**	293	0.2705 (6e-08)
**4**	62	0.1747 (5e-04)
**5**	26	0.1437 (4e-03)

Table S 7. The significant correlation between cross-talk degree and binary module essentiality persists for a range of odd-scores in the *Pull-down* network for (a) protein complexes and (b) filtered biological processes. Odd-score cutoff gives the minimum odd-score for a module pair to be considered a cross-talk. **# Cross-talks** gives the number of cross-talks at each cutoff. **SRCC (p-value)** gives the Spearman's rho rank correlation coefficient between cross-talk degree and binary module essentiality. For larger odd-score cutoffs, there are fewer cross-talks and this results in somewhat lower SRCC values.
