# Supplementary material for: From Hub Proteins to Hub Modules: The Relationship Between Essentiality and Centrality in the Yeast Interactome at Different Scales of Organization
Source: PLoS Comput Biol. 2013 Feb 21;9(2):e1002910. doi: 10.1371/journal.pcbi.1002910 (PMC3578755; doi:10.1371/journal.pcbi.1002910)
Supplement: Table S8 — The significant correlation between cross-talk degree and binary module essentiality persists for a range of odd-scores in the Full network. (PDF) [file pcbi.1002910.s025.pdf]

(a) Protein complexes

Odd-score cutoff	#Cross-talks	SRCC (p-value)
**1.5**	910	0.4145 (0)
**2**	727	0.4081 (0)
**3**	428	0.3805 (7e-15)
**4**	245	0.3139 (2e-10)
**5**	154	0.2794 (2e-08)

(b) Filtered biological processes

Odd-score cutoff	#Cross-talks	SRCC (p-value)
**1.5**	4545	0.3974 (3e-16)
**2**	2306	0.3606 (2e-13)
**3**	470	0.2753 (3e-08)
**4**	116	0.1055 (4e-02)
**5**	45	0.1074 (3e-02)

Table S 8. The significant correlation between cross-talk degree and binary module essentiality persists for a range of odd-scores in the *Full* network for (a) protein complexes and (b) filtered biological processes. Odd-score cutoff gives the minimum odd-score for a module pair to be considered a cross-talk. **# Cross-talks** gives the number of cross-talks at each cutoff. **SRCC (p-value)** gives the Spearman's rho rank correlation coefficient between cross-talk degree and binary module essentiality. For larger odd-score cutoffs, there are fewer cross-talks and this results in somewhat lower SRCC values.
