# Supplementary material for: From Hub Proteins to Hub Modules: The Relationship Between Essentiality and Centrality in the Yeast Interactome at Different Scales of Organization
Source: PLoS Comput Biol. 2013 Feb 21;9(2):e1002910. doi: 10.1371/journal.pcbi.1002910 (PMC3578755; doi:10.1371/journal.pcbi.1002910)
Supplement: Table S9 — The number of proteins, the number of interactions and the fraction of essential proteins for the Y2H-union and BinaryHQHT physical interaction networks. (PDF) [file pcbi.1002910.s026.pdf]

Network	Num Proteins	Num Interactions	Fraction of essential proteins
**Y2H-union**	1815	2478	0.24
**BinaryHQHT**	3063	7428	0.21

Table S 9. The number of proteins, the number of interactions and the fraction of essential proteins for the *Y2H-union* and *BinaryHQHT* physical interaction networks.
