# Supplementary material for: From Hub Proteins to Hub Modules: The Relationship Between Essentiality and Centrality in the Yeast Interactome at Different Scales of Organization
Source: PLoS Comput Biol. 2013 Feb 21;9(2):e1002910. doi: 10.1371/journal.pcbi.1002910 (PMC3578755; doi:10.1371/journal.pcbi.1002910)
Supplement: Table S11 — A substantial fraction of physical interactions in the BinaryHQHT network are intermodular. (PDF) [file pcbi.1002910.s028.pdf]

Modules	Num Annotated Interactions	Num Intramodular Interactions	Num Intermodular Interactions	Intermodular Percentage
Protein complexes	1416	399	1017	71.82%
BPs, 50	4565	716	3849	84.33%
Filtered BPs	2050	196	1854	90.44%

Table S 11. A substantial fraction of physical interactions are intermodular in the *BinaryHQHT* network. Modules gives the set of functional modules considered. These are: 1) protein complexes, 2) a subset of specific GO BP terms, each of which annotates at most 50, 100, 300, or 500 proteins in the yeast genome, or 3) a subset of filtered biological processes (as decribed in **Materials and Methods**). **Num Annotated Interactions** gives the number of interactions in the subnetwork generated from the *BinaryHQHT* network where nodes represent proteins in the considered modules and edges represent interactions amongst them. **Num Intramodular Interactions** gives the number of interactions in the subnetwork where the two interacting proteins belong to the same module. **Num Intermodular Interactions** gives the number of interactions in the subnetwork where the two interacting proteins belong to different modules. **Intermodular Percentage** gives the percentage of intermodular interactions amongst the annotated interactions.
