# Supplementary material for: From Hub Proteins to Hub Modules: The Relationship Between Essentiality and Centrality in the Yeast Interactome at Different Scales of Organization
Source: PLoS Comput Biol. 2013 Feb 21;9(2):e1002910. doi: 10.1371/journal.pcbi.1002910 (PMC3578755; doi:10.1371/journal.pcbi.1002910)
Supplement: Table S12 — Within each essential protein complex, essential proteins tend to have a higher average intracomplex degree in Y2H-union and BinaryHQHT networks. (PDF) [file pcbi.1002910.s029.pdf]

Network	Num Complexes Tested	Num Complexes with Higher Avg Essential Degree	Empirical p-value
Y2H-union	13	11 (84.62%)	7e-3
**BinaryHQHT**	14	11 (78.57%)	2e-2

Table S 12. Within each essential protein complex, essential proteins tend to have a higher average intracomplex degree. Num Complexes Tested gives the number of complexes considered; each has at least two essential proteins and at least two non-essential proteins with intracomplex interactions. **Num Complexes with Higher Avg Essential Degree** gives the number of complexes among the tested complexes where essential proteins have a higher intracomplex degree on average than non-essential proteins. To compute the **Empirical p-value**, we did 10,000 random permutations of protein essentiality assignment, and for each permutation we computed the number of complexes with higher average degree for its essential proteins. The p-value is the fraction of random permutations where the number of such complexes is greater than or equal to the actual number, computed over the 10,000 random permutations.
