# Supplementary material for: From Hub Proteins to Hub Modules: The Relationship Between Essentiality and Centrality in the Yeast Interactome at Different Scales of Organization
Source: PLoS Comput Biol. 2013 Feb 21;9(2):e1002910. doi: 10.1371/journal.pcbi.1002910 (PMC3578755; doi:10.1371/journal.pcbi.1002910)
Supplement: Table S13 — Module-level networks for Y2H-union and BinaryHQHT networks. (PDF) [file pcbi.1002910.s030.pdf]

(a) Protein Complexes

Network	Num Cross-talks	Num Modules	Fraction of Essential Modules
Y2H-union	2	4	0.75
BinaryHQHT	46	54	0.81

(b) Filtered Biological Processes

Network	Num Cross-talks	Num Modules	Fraction of Essential Modules
Y2H-union	49	62	0.92
BinaryHQHT	618	224	0.82

Table S 13. Module-level networks for (a) protein complexes and (b) filtered biological processes. A module-level network was built for (a) protein complexes and (b) filtered biological processes using each of the networks. **Num Cross-talks** gives the number of inferred cross-talks. **Num Modules** gives the number of modules with at least one inferred cross-talk. **Fraction of Essential Modules** gives the fraction of modules having at least one essential protein, amongst modules with at least one cross-talk.
