# Supplementary material for: From Hub Proteins to Hub Modules: The Relationship Between Essentiality and Centrality in the Yeast Interactome at Different Scales of Organization
Source: PLoS Comput Biol. 2013 Feb 21;9(2):e1002910. doi: 10.1371/journal.pcbi.1002910 (PMC3578755; doi:10.1371/journal.pcbi.1002910)
Supplement: Table S14 — Numbers of bait proteins in the Pull-downf network. (PDF) [file pcbi.1002910.s031.pdf]

Network	# Proteins	# Interactions	# Bait proteins	# Bait intramodular interactions	# Bait intermodular interactions
**Pull-down^f**	4120 (0.23)	30877	2708 (0.29)	3833	13958

Table S 14. Bait analysis. For the *Pull-down* network, we exclude interactions from small-scale experiments (< 50 interactions determined). We call this network *Pull-down^f*. This network will be utilized to make comparisons amongst bait proteins only, as bait proteins may be enriched in essential proteins and in interactions. The total number of proteins, the total number of interactions, the number of bait proteins, and the number of intramodular and intermodular interactions involving bait proteins is given. The fraction of essential proteins in each network is shown within the parentheses next to the number of proteins.
