# Supplementary material for: From Hub Proteins to Hub Modules: The Relationship Between Essentiality and Centrality in the Yeast Interactome at Different Scales of Organization
Source: PLoS Comput Biol. 2013 Feb 21;9(2):e1002910. doi: 10.1371/journal.pcbi.1002910 (PMC3578755; doi:10.1371/journal.pcbi.1002910)
Supplement: Table S15 — Within each essential protein complex, essential bait proteins tend to have a higher average intracomplex degree than non-essential bait proteins in the Pull-downf network. (PDF) [file pcbi.1002910.s032.pdf]

	Num Complexes Tested	Num Complexes with Higher Avg Essential Degree	Empirical p-value
Pull-down[f]	42	28 (66.67%)	0e+00

Table S 15. Within each essential protein complex, essential bait proteins tend to have a higher average intracomplex degree than non-essential bait proteins. Num Complexes Tested gives the number of complexes considered in each of the two networks excluding small-scale experiments; each such complex was required to have at least two essential bait proteins and at least two non-essential bait proteins, each with intracomplex interactions. **Num Complexes with Higher Avg Essential Degree** gives the number of complexes among the tested complexes where the essential bait proteins have higher intracomplex degree on average than the non-essential bait proteins. To determine whether this number is significant, we randomly permuted essentiality assignments and computed the number of complexes with higher average intracomplex degrees for essential bait proteins. **Empirical p-value** gives the fraction of random permutations where the number of such complexes is greater than or equal to the actual number, computed over 10,000 permutations.
