# Supplementary material for: From Hub Proteins to Hub Modules: The Relationship Between Essentiality and Centrality in the Yeast Interactome at Different Scales of Organization
Source: PLoS Comput Biol. 2013 Feb 21;9(2):e1002910. doi: 10.1371/journal.pcbi.1002910 (PMC3578755; doi:10.1371/journal.pcbi.1002910)
Supplement: Text S1 — Analysis on the correlations between interaction degree and essentiality for proteins in all complexes, including ribosomal complexes, for all three networks. (PDF) [file pcbi.1002910.s033.pdf]

The four complexes pertaining to the ribosome tend to be removed from analysis in the literature [1,2] because they have a relatively large number of member proteins (ranging from 32 to 79) yet have a low fraction of essential proteins ranging from 4.55% to 15.19%. Due to the total number of proteins, computational analysis can be largely affected by these four complexes. We still observe a large difference in the correlation between essentiality and intracomplex interaction degree, as well as essentiality and all interaction degree when the four ribosomal complexes are included (Figure S6 (a)). Including these four complexes does not affect our finding much in the *Direct* network (Figure S6 (a) vs. Figure 2 (a)), but it has a larger effect in the two other networks (Figures S6 (b) and (c) vs. Figures S4 (a) and S5 (a)). This may be due to the fact that the other networks contain indirect interactions, and non-essential proteins within large complexes can have a larger number of intracomplex interactions than essential proteins within small complexes. In particular, if there are many indirect interactions, the large complexes may have a higher chance to have many indirect intracomplex interactions than small complexes. Throughout the main body of the paper, we removed these ribosomal complexes for the reported complexes, unless otherwise noted.

References

1. Hart GT, Lee I, Marcotte EM (2007) A high-accuracy consensus map of yeast protein complexes reveals modular nature of gene essentiality. BMC Bioinformatics 8: 236.

2. Wang H, Kakaradov B, Collins SR, Karotki L, Fiedler D, et al. (2009) A complex-based reconstruction of the Saccharomyces cerevisiae interactome. Molecular and Cellular Proteomics 8: 1361–1381.
